# Sulfate starvation response modules connect sulfur metabolism to photorespiration and photosynthesis

**DOI:** 10.1111/tpj.71019

**Published:** 2026-07-09

**Authors:** Suvajit Basu, Inken Thiemann, Stefan Timm, Varsa Shukla, Philipp Baessler, Philipp Westhoff, Stanislav Kopriva, Daniela Ristova

**Affiliations:** ^1^ Institute for Plant Sciences, University of Cologne 50674 Cologne Germany; ^2^ Plant Physiology Department University of Rostock D‐18051 Rostock Germany; ^3^ Plant Metabolism and Metabolomics Facility Heinrich Heine University Düsseldorf 40225 Germany; ^4^ Cluster of Excellence on Plant Sciences (CEPLAS) Cologne Germany

**Keywords:** sulfate, starvation, transcription, photorespiration, photosynthesis

## Abstract

Mineral nutrients are essential for plant growth and development. Sulfur (S), as a macronutrient, is incorporated into numerous critical S‐containing metabolites that play key roles in mitigating both abiotic and biotic stresses. Understanding how plants regulate S homeostasis and integrate it with other physiological processes is crucial for developing crops that can better withstand environmental challenges. Here, we demonstrate that the conserved sulfate starvation transcriptional response across Arabidopsis, tomato, rice, and *Setaria* is limited to only seven genes. We further characterize the roles of two of these genes, *PYD4* (*PYRIMIDINE 4*) and *MGL* (*METHIONINE GAMMA‐LYASE*), in S metabolite regulation and the sulfate starvation response. Our genetic and biochemical analyses show that PYD4 is embedded within the S starvation network, positively regulating transcript levels of key S‐marker genes, including four of the seven conserved genes across these species, and serving as an integrator of S metabolism and photorespiration. MGL also positively regulates S‐marker genes, while additionally modulating diverse processes under sulfate starvation, such as photosynthesis and oxidoreductase homeostasis. Notably, MGL‐deficient lines fail to respond adequately to sulfate starvation and exhibit impaired mechanisms for maintaining photosynthetic efficiency. Overall, our findings indicate that the S starvation response is deeply embedded within primary plant metabolism. Disruption of its regulators alters metabolism at multiple levels, affecting traits central to crop improvement, such as photorespiration and photosynthesis.

## Significance Statement

How plants regulate mineral homeostasis and integrate it with other physiological processes is crucial for developing crops that can better withstand environmental challenges. In this study, we show that *PYD4* (*PYRIMIDINE 4*) and *MGL* (*METHIONINE GAMMA‐LYASE*), are involved in sulfate starvation response regulation. Moreover, PYD4 is critical for mediating photorespiration under sulfur deficiency, whereas MGL activity during sulfate starvation interfaces with multiple processes, such as maintaining photosynthetic efficiency.

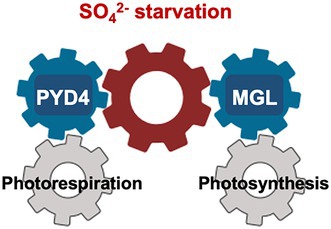

## INTRODUCTION

Plants, as sessile organisms, continuously face environmental fluctuations and have developed sophisticated mechanisms to perceive and adapt to these changes, including variations in nutrient availability. Modern agricultural practices, such as the widespread application of fertilizers, have advanced crop yields but also contributed to significant global environmental repercussions, including biodiversity loss, excessive mining and depletion of macronutrients, and climate change (DeLoose et al., 2024; Fowler et al., 2013). Furthermore, climate change‐manifested by rising temperatures, increased drought, and higher atmospheric CO_2_ concentrations negatively impacts vegetation, leading to reduced nutrient content and nutrient use efficiency in plants (Querejeta et al., 2021; Terrer et al., 2019).

Research investigating nutrient signaling in plants has mainly focused on nitrogen (N) and phosphorus (P), given their critical roles in determining crop yield (Bouain et al., 2019). In contrast, signaling networks regulating sulfur (S) homeostasis remain less explored (Ristova & Kopriva, 2022). Recent research demonstrates that sulfur‐containing metabolites and cofactors are pivotal in counteracting abiotic and biotic challenges, including sustaining redox equilibrium under stress conditions (Kumar et al., 2019; Bonnot et al., 2020; Henriet et al., 2021), enhancing tolerance and detoxification of soilborne toxic metals (Gill & Tuteja, 2011; Sun et al., 2021, 2023), and mediating defense responses against pathogen infection and herbivory (Gigolashvili et al., 2007). The role of S nutrition thus extends to plant resilience and even influences host‐microbiome interactions, which can affect health and disease in both plants and their associated organisms (D'Agostino et al., 2024).

Plant roots uptake sulfate (SO_4_^2−^) from the soil and transport it to the shoots via the xylem, where it is assimilated and incorporated into cysteine (Cys). Cys is subsequently utilized for protein synthesis, glutathione (GSH) production, or metabolized to methionine (Met), and can donate sulfur atoms to a wide variety of essential molecules and specialized metabolites (Kopriva et al., 2012). While the sensor responsible for the sulfate starvation response remains unidentified (Ristova & Kopriva, 2022), the key transcriptional regulator, SULFUR LIMITATION1 (SLIM1)/ETHYLENE‐INSENSITIVE3‐LIKE3 (EIL3), has been characterized (Maruyama‐Nakashita et al., 2006). SLIM1/EIL3 orchestrates the induction of sulfate transporters, activation of sulfate uptake, and degradation of glucosinolates (GSLs) during sulfate starvation in *Arabidopsis thaliana* (Dietzen et al., 2020; Maruyama‐Nakashita et al., 2006). The upregulation of sulfate transporters, assimilation genes, and regulators of S homeostasis and catabolism of GSH and GSLs depends on the SLIM1/EIL3 pathway, and this response is attenuated in *slim1* or *eil3* mutants (Dietzen et al., 2020; Maruyama‐Nakashita et al., 2006; Ristova & Kopriva, 2022). While SLIM1/EIL3 transcript and protein levels remain unchanged by sulfate starvation in Arabidopsis, crop species such as tomato (*Solanum lycopersicum*) (Canales et al., 2020), rice (*Oryza sativa* Kitaake), and setaria (*Setaria viridis*) (Zenzen et al., 2024) exhibit marked *SLIM1* transcript upregulation in shoots and roots, indicating species‐specific regulatory mechanisms and suggesting the existence of additional conserved regulators.

In this study, we reveal that only seven genes constitute the transcriptional sulfate starvation response shared among Arabidopsis, tomato (*S. lycopersicum*), rice (*O. sativa*), and setaria (*S. viridis*), mostly regulated by SLIM1/EIL3 in Arabidopsis. We focus on the roles of two of these intersecting genes, *PYD4* (At3g08860) and *MGL* (At2g64660), which are vital for S metabolism and homeostasis. Analysis of independent allele lines demonstrates that S‐containing metabolites, including thiols and GSLs, as well as the expression of S starvation sentinel genes, are affected in the respective loss‐of‐function mutants. We further show that PYD4 is critical for mediating photorespiration under sulfur starvation, whereas MGL activity during sulfate starvation interfaces with multiple processes, including photosynthesis, oxidoreductase balance, and secondary metabolite production. Taken together, these seven genes represent integral components of the plant sulfate starvation network, with PYD4 and MGL playing established roles in Arabidopsis and likely in other crop species as well.

## RESULTS

### Only seven genes are differentially regulated under sulfate starvation across four different plant species

**Figure 1:**
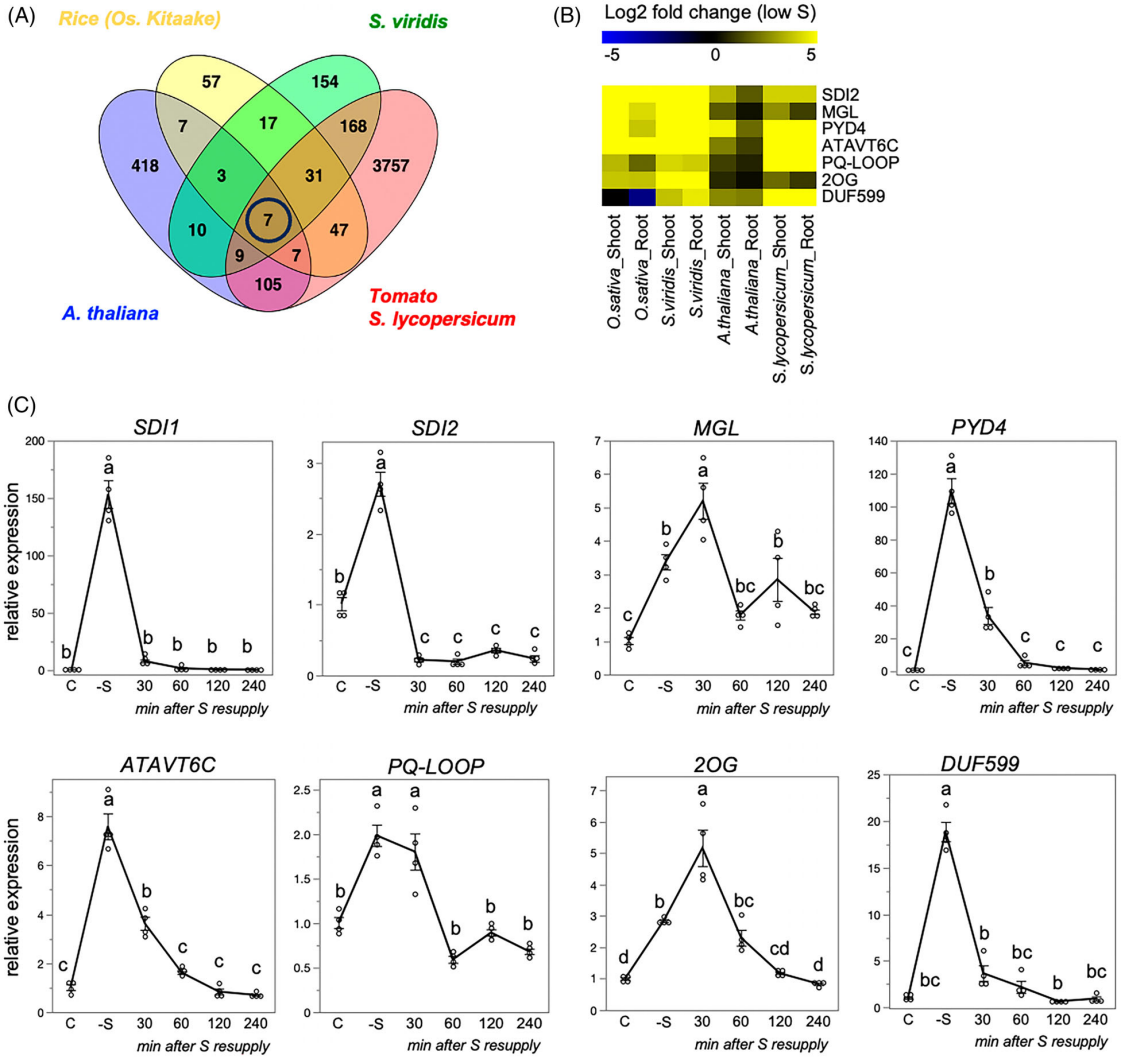
Seven shared genes differentially upregulated under sulfate starvation in two monocots and two dicots, and their transcript downregulation after sulfate resupply in Arabidopsis. (A) Intersect between differentially expressed genes (DEGs) regulated by sulfate starvation in four plant species: two monocots, *Setaria viridis* and *Oryza sativa* Kitaake (Zenzen et al., 2024); and two dicots, *Arabidopsis thaliana* and *Solanum lycopersicum* (tomato) (Canales et al., 2020; Dietzen et al., 2020). (B) Heatmap of expression (log2 FC) under sulfate starvation of the seven intersecting genes in roots and shoots in the four species (*O.sativa*—*O*. *sativa* Kitaake; *S.viridis*—*Setaria viridis*; *A.thaliana*—*A. thaliana*; and *S*.*lycopersicum*—*Solanum lycopersicum*). (C) Relative expression of the intersect genes (including *SDI1*) in *A. thaliana* roots after resupply of sulfate in plants grown under sulfate starvation (‐S), including plants grown on full media (C) for 14 days. Plants were grown hydroponically on full media [0.75 mM SO_4_^2−^] or low sulfate media [0.015 mM SO_4_^2−^] to achieve transcriptional upregulation followed by resupply of sulfate [0.75 mM SO_4_^2−^] to the S‐deficient plants, and harvest after 30, 60, 120, and 240 min. Relative gene expression was determined via reverse transcription quantitative PCR (RT‐qPCR) normalized to two housekeeping genes. Means are given with standard errors. Different letters indicate statistically significant difference for one‐way anova, and Tukey's HSD test for multiple comparisons (*n* = 4). *MGL, METHIONINE GAMMA‐LYASE; PYD4, PYRIMIDINE 4; SDI1/SDI2, SULFUR DEFICIENCY‐INDUCED1/2; ATAVT6C, AT3G56200; PQ‐LOOP, AT5G40670; 2OG, AT3G19000; DUF599, AT4G31330*.

In recent work, we showed that transcriptional response to sulfate starvation in two monocots, *O. sativa* Kitaake and *S. viridis*, is largely species‐specific, and only 58 differentially expressed genes (DEGs) are common between the two species (Zenzen et al., 2024). To identify novel regulators of sulfate starvation response, relevant for Arabidopsis and other plant species, we intersected shared DEGs from the two monocots (Zenzen et al., 2024), with DEGs regulated by sulfate starvation in two dicot plants, tomato (*S. lycopersicum*) (Canales et al., 2020), and Arabidopsis (Dietzen et al., 2020). Only seven genes were shared between the four species (Figure 1A), namely, *SULFUR DEFICIENCY‐INDUCED 2* (*SDI2, At4g04770*), *METHIONINE GAMMA‐LYASE* (*MGL, At2g64660*), *PYRIMIDINE 4* (*PYD4, At3g08860*), 2‐oxoglutarate (2OG) and Fe (II)‐dependent oxygenase (*At3g19000*), two genes for transmembrane proteins predicted to localize to the vacuole (Hooper et al., 2017) (*ATAVT6C*, *At3g56200*; and *PQ‐loop, At5g40670*), and a gene for transmembrane protein predicted to localize to the plasma membrane (Hooper et al., 2017) (*DUF599*, *At4g31330*). Most of these genes were upregulated in both roots and shoots under sulfate starvation in the four plant species (Figure 1B).

Remarkably, the functional roles of these seven genes in sulfate starvation response and broader S metabolism remain largely unexplored except for *SDI1*/*SDI2*. The *SDI* genes are negative regulators of GSL synthesis in Arabidopsis and are highly upregulated under sulfate starvation (Aarabi et al., 2016). Additionally, *SDI* genes reduce sulfur‐rich 2S seed storage proteins and sulfolipids under sulfate starvation (Aarabi et al., 2021, 2023). *SDI1* and *SDI2* belong to the ‘OAS cluster’, a group of six genes that shows high correlation with OAS (O‐acetylserine) accumulation (Hubberten et al., 2012). Overall, our findings emphasize that, despite the taxonomic breadth, only a minimal set of seven genes underlies the conserved transcriptional S starvation response in plants, highlighting potentially new regulatory axes for S homeostasis.

### The intersect genes are upregulated by sulfate starvation and repressed after S resupply

To verify that upregulation of the seven conserved sulfate starvation‐responsive genes depends on sulfate availability, we evaluated their transcript levels following sulfate resupply. Previous studies have shown that the expression of most sulfate starvation marker genes—upregulated under S deficiency—rapidly returns to baseline steady‐state levels after sulfate is reintroduced to the growth medium (Bielecka et al., 2014). To quantify the regulatory dynamics, Arabidopsis plants were grown hydroponically under low sulfur conditions for 14 days, then supplied with sulfate, and transcript abundance monitored at 30, 60, 120, and 240 min post‐resupply. Full media‐grown controls were used for comparison. Both *SDI1* and *SDI2* were included since crop species typically possess only a single SDI ortholog (Zenzen et al., 2024). Our analysis revealed that *SDI1* and *DUF599* transcript levels returned to steady‐state levels after only 30 min of sulfate resupply, indicating rapid transcript degradation. Notably, *SDI2* expression also decreased at 30 min, but dropped below control levels (Figure 1C). *PYD4* and *ATAVT6C* normalized by 60 min, with *PQ‐LOOP* remaining upregulated at 30 min before returning to control levels at 60 min. In contrast, *MGL* and *2OG (At3g19000)* transcripts showed an initial increase at 30 min followed by normalization at 60–120 min. Overall, these results demonstrate that prolonged sulfur deficiency upregulates all seven genes in Arabidopsis roots, while sulfate resupply leads to a rapid return to baseline expression levels, typically within 30–120 min. This reinforces the notion that their transcriptional regulation is tightly and dynamically linked to sulfate status in the plant.

### SLIM1/EIL3 regulates all seven shared genes, completely or partially

**Figure 2:**
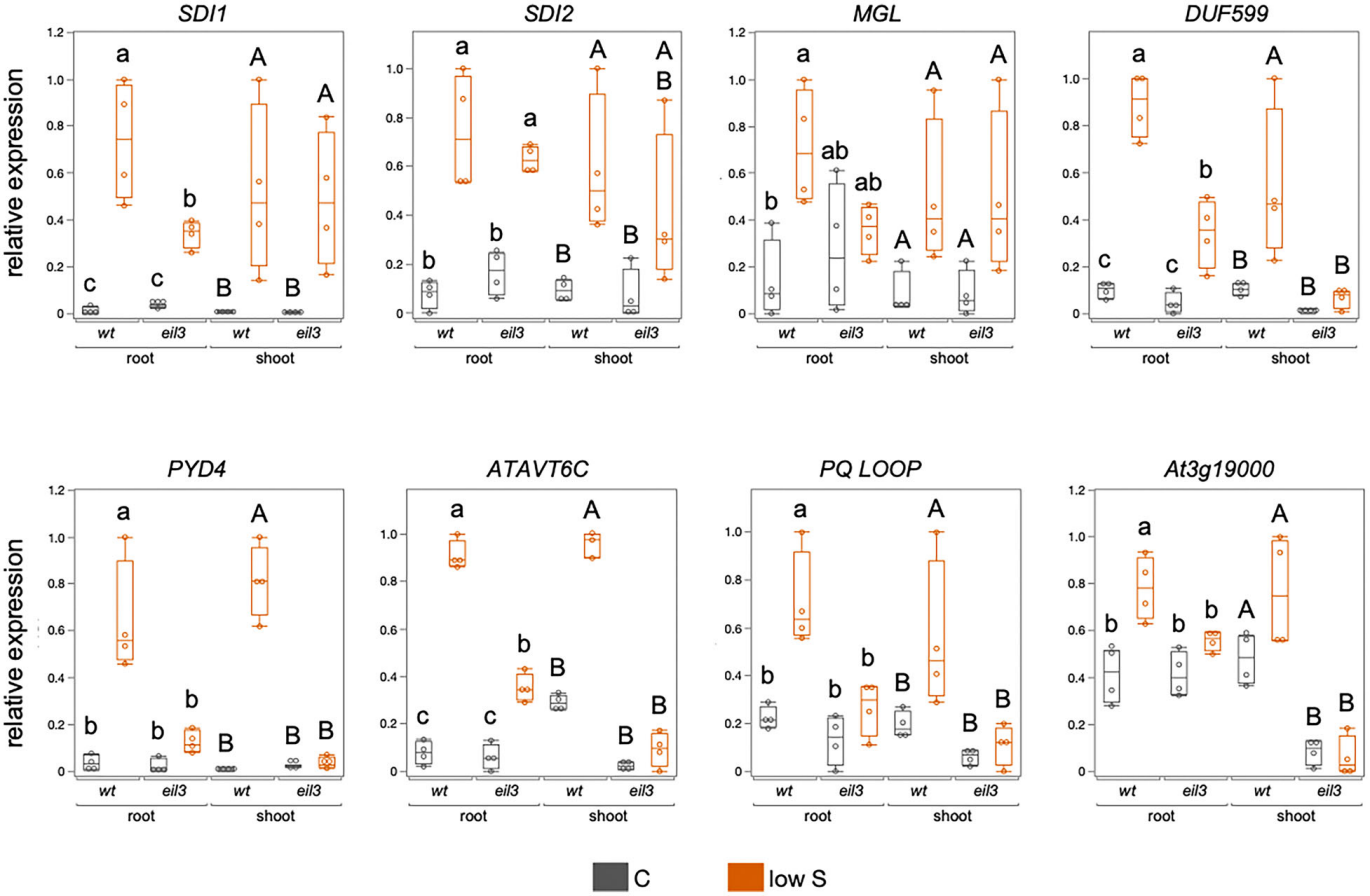
Shared genes differentially upregulated under sulfate starvation in roots and shoots, and their transcript regulation in *eil3* mutant. Relative expression of the intersect genes in *Arabidopsis thaliana* wild‐type (*wt*) and *eil3* mutant roots and shoots. Data are means ± SE; *n* = 4 biological replicates. Significant different changes between the genotypes and conditions are determined using two‐way anova and Tukey's HSD test for multiple comparisons for each organ. Lower letters refer to statistical significance for the root, while capital letters for the shoot. Absence of letters indicates no statistical differences. Plants were grown on plates for 18 days on either control [0.75 mM SO_4_^2−^] (C) or low sulfate media [0.015 mM SO_4_^2−^] (low S). Relative gene expression of eight genes was determined via reverse transcription quantitative PCR (RT‐qPCR) normalized to two housekeeping genes, and scaled (0–1) for side‐by‐side visualization. *MGL, METHIONINE GAMMA‐LYASE; PYD4, PYRIMIDINE 4; SDI1/SDI2, SULFUR DEFICIENCY‐INDUCED1/2; ATAVT6C, AT3G56200; PQ‐LOOP, AT5G40670; 2OG, AT3G19000; DYF599, AT4G31330*.

SLIM1/EIL3 has been established as a central transcriptional regulator of the plant sulfate starvation response, acting upstream of core sulfur metabolism pathways in Arabidopsis. Recently, we have identified core genes that are under control of SLIM1/EIL3 (Ristova & Kopriva, 2022), based on two transcriptomic studies (Dietzen et al., 2020; Maruyama‐Nakashita et al., 2006). Four of the seven intersecting genes (*SDI1, PYD4, PQ‐LOOP, DUF599*) belong to the cluster of genes that are upregulated under S deficiency, but the upregulation is significantly diminished or abolished in the *slim1/eil3* mutant background (Ristova & Kopriva, 2022). To evaluate the direct contribution of SLIM1/EIL3 to the regulation of these seven genes, Arabidopsis wild‐type (WT) and *eil3* mutant plants were grown on agar plates under full nutrient and low sulfate media conditions, as previously described (Dietzen et al., 2020). Gene expression was quantified in both roots and shoots for all seven genes, with *SDI1* included alongside *SDI2* due to genetic redundancy. As expected, all genes were significantly upregulated in the WT under S deficiency, in both roots and shoots, except for *MGL* and *2OG (At3g19000)* in shoots (Figure 2). In contrast, the upregulation for *PYD4* and the three transporters (*DUF599, ATAVT6C, PQ‐LOOP*) was attenuated in both roots and shoots of *eil3* mutants, while *SDI1* showed an attenuated response only in roots. Also, *MGL* was induced in the roots of WT plants in response to S deficiency, but this induction was absent in the *eil3* background, while this was similar for *SDI2* but in the shoots (Figure 2). These results indicate that SLIM1/EIL3 regulates all intersecting sulfur‐deficiency‐responsive genes in Arabidopsis, albeit with varying strength. Notably, its regulatory effect is confined to the roots for *SDI1* and *MGL*, and to the shoots for *SDI2*. This also suggests that additional, yet uncharacterized, regulatory mechanisms participate in the broader transcriptional sulfate starvation response in plants.

### Involvement of MGL and PYD4 in S homeostasis

**Figure 3:**
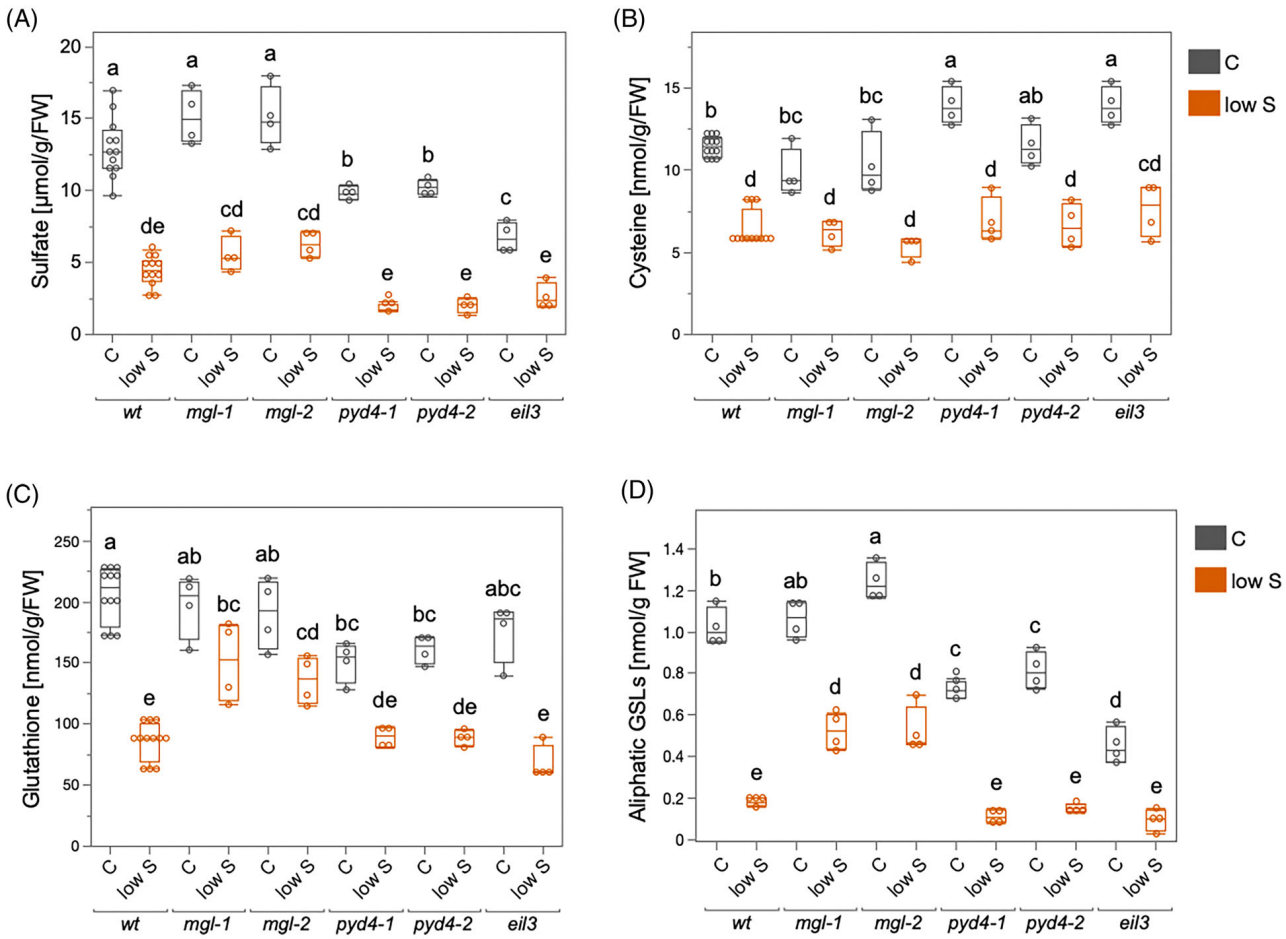
Mutation of MGL or PYD4 impact sulfate homeostasis. (A) Quantification of sulfate anions (μmol g^−1^ FW) in shoots of six genotypes under full media (C) and low sulfate media (low S). (B) Quantification of cysteine (μmol g^−1^ FW) of six genotypes under full media and low sulfate (low S). (C) Quantification of glutathione (μmol g^−1^ FW) of six genotypes under full media and low sulfate (low S). (D) Quantification of aliphatic glucosinolates (μmol g^−1^ FW) of six genotypes under full media (C) and low sulfate (low S). Data are means ± SE; *n* = 4 biological replicates. Different letters indicate statistically significant difference for two‐way anova and Tukey's HSD test for multiple comparisons. Plants were grown on vertical plates for 18 days under full media (0.75 mM SO_4_^2−^) or low sulfate media (0.015 mM SO_4_^2−^), and shoots were used for quantification. Ten plants were plated on each plate and, when harvesting, 10 shoots from each plate were bulked into one sample. Genotypes: Col‐0 (*wt*), *mgl‐1*, *mgl‐2*, *pyd4‐1*, *pyd4‐2*, and *eil3* (Dietzen et al., 2020).

Since the roles of the shared genes in the sulfate starvation response are unknown, we selected two for detailed analysis: *PYD4*, which is under SLIM1/EIL3 control, and *MGL*, which is regulated by SLIM1/EIL3 only in the roots. Two independent knockdown/knockout mutants were obtained for each gene (designated *pyd4‐1*, *pyd4‐2, mgl‐1*, and *mgl‐2*) (Figure S1). *MGL* encodes a cytosolic enzyme that catalyzes the degradation of methionine into methanethiol, alpha‐ketobutyrate, and ammonia (Goyer et al., 2007), and is upregulated under sulfate starvation (Dietzen et al., 2020). *PYD4* expression is also upregulated under sulfate starvation (Dietzen et al., 2020; Maruyama‐Nakashita et al., 2006), but additionally under osmotic stress (Winter et al., 2007). Mutants in regulators of sulfate starvation response often have altered S homeostasis and altered levels of sulfate anions, thiols, and S‐rich secondary metabolites, such as GSLs (Aarabi et al., 2016; Dietzen et al., 2020; Maruyama‐Nakashita et al., 2006). Therefore, we measured shoot sulfate content in mutants including *eil3*, grown on vertical plates. As expected, sulfate levels dropped by 60–80% under sulfate starvation in the WT (Dietzen et al., 2020), with both *pyd4* mutants showing a lower sulfate content than the WT in full media (Figure 3A). A similar sulfate phenotype was observed in *eil3*, consistent with previous observations (Dietzen et al., 2020; Kawashima et al., 2011). Nitrate and phosphate anions remained unchanged, suggesting a specific disruption of sulfate (Figure S2A,B).

We then quantified Cys and GSH in the shoots. No differences in Cys accumulation were observed (Figure 3B). However, both *mgl‐1* and *mgl‐2* lines showed significantly lower decreases in accumulation of GSH in response to S deficiency, leading to absent or very small decline in GSH in response to sulfate starvation compared with WT (Figure 3C). In contrast, compared with WT, both *pyd4‐1* and *pyd4‐2* displayed lower GSH levels under full nutrient conditions, but not under sulfate starvation, resembling the phenotype of *eil3* and *slim1‐1* (Figure 3A) (Kawashima et al., 2011). Taken together, these findings suggest distinct roles for PYD4 and MGL in S metabolism, where PYD4‐deficient lines are altered under steady‐state, while MGL‐deficient lines are affected primarily in sulfate starvation conditions, suggesting different mechanisms of action.

GSLs are sulfur‐rich secondary metabolites that harbor anti‐pathogenic and anti‐herbivory plant‐protective functions and have medicinal properties (Halkier & Gershenzon, 2006). During sulfate starvation GSL biosynthesis is repressed, and their catabolism is stimulated ensuring recycling of S pools (Sugiyama et al., 2021). Given the disruption of S primary metabolism in MGL and PYD4‐deficient mutants, we hypothesized that GSLs accumulation could also be affected. Indeed, under full nutrient conditions, the *pyd4* lines displayed significantly lower levels of aliphatic GSLs, consistent with patterns observed earlier for sulfate and GSH content (Figure 3D; also Figure 3A,C). In contrast, both *mgl* lines exhibited diminished decrease in aliphatic GSLs in response to sulfate starvation, comparably to GSH (Figure 3C). No significant differences were observed in indolic GSL content across all genotypes (Figure S2C), suggesting that MGL and PYD4 are mainly acting on Met‐derived GSLs pathway. The decrease in aliphatic GSLs in *pyd4* mutants under control conditions mirrors the phenotype seen in *eil3* mutants (Dietzen et al., 2020), indicating that *pyd4* mutants phenocopy *eil3* with respect to both GSL and primary S metabolite profiles. Taken together, these results support the conclusion that both MGL and PYD4 activities are essential for the correct synthesis and/or catabolism of aliphatic GSLs, which plays a crucial role in facilitating sulfur recycling under fluctuating environmental sulfur supply.

### MGL and PYD4 are required for increased flux through sulfate assimilation during sulfate starvation

**Figure 4:**
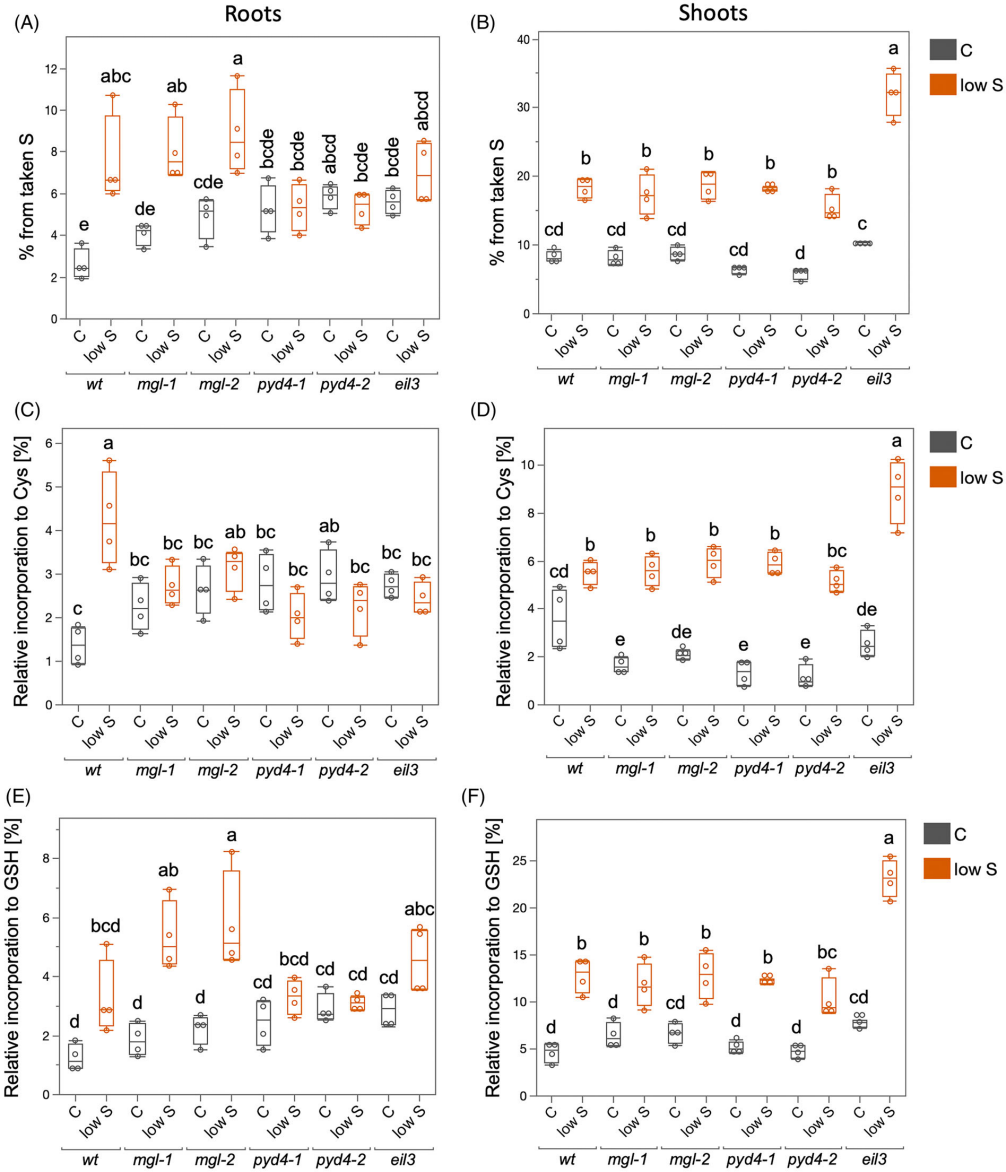
Mutation of MGL or PYD4 impacts thiol flux. (a) Quantification of relative Cys (cysteine) incorporation (flux) in six genotypes under C and low S in roots. (b) Quantification of relative sulfate incorporation (flux) in six genotypes under C and low S in shoots. (c) Quantification of relative sulfate incorporation (flux) in six genotypes under C and low S in roots. (d) Quantification of relative Cys (cysteine) incorporation (flux) in six genotypes under C and low S in shoots. (e) Quantification of relative GSH (glutathione) incorporation (flux) in six genotypes under C and low S in roots. (f) Quantification of relative GSH (glutathione) incorporation (flux) in six genotypes under C and low S in shoots. Data are mean ± SE (*n* = 4 biological replicates). Different letters indicate statistically significant difference for two‐way ANOVA, and Tukey HSD test for multiple comparisons. Arabidopsis plants (25 seedlings) were grown on a nylon net in hydroculture for 14 days under full‐media (C, 0.75 mM SO_4_^2−^) and low sulfate media (low S, 0.015 mM SO_4_^2−^), and thereafter incubated with [35S] sulfate for 4 h. Shoots and roots were harvested separately and thiols extracted. 35S incorporation to Cys and GSH was measured via HPLC with a radio‐detector. Genotypes: *Col‐0* (wt), *mgl‐1*, *mgl‐2*, *pyd4‐1*, *pyd4‐2*, and *eil3* (Dietzen et al., 2020).

To evaluate whether the loss of MGL and PYD4 also affects the flux through sulfate assimilation, the mutants and the previously described *eil3* (Dietzen et al., 2020) were grown under full media and sulfate starvation media for 2 weeks and subsequently fed with [^35^S]sulfate for 4 h. The incorporation of ^35^S into thiols, in both roots and shoots, was determined. In the roots, sulfate starvation caused increased flux in WT and the *mgl* mutants, whereas it did not affect the flux in *pyd4* and *eil3* (Figure 4A). In shoots, the flux through sulfate assimilation was again induced by sulfate starvation in the same manner in WT and the mutants, but in *eil3*, this increase was even greater (Figure 4B). Interestingly, when ^35^S incorporation in the individual thiols was compared, a more complex pattern occurred. Although the total flux in the roots was the same in WT and *mgl* lines, the partitioning of ^35^S was different. In WT roots, the incorporation into Cys was increased by sulfate starvation, whereas in *mgl* and the *pyd4* mutants, the incorporation into Cys remained the same in both conditions, whereas the labeling of GSH in *mgl* increased in sulfate starvation compared with control to a greater level than in WT (Figure 4C,E). In the WT shoots, sulfate starvation triggered a greater increase in ^35^S incorporation into GSH (2.75‐fold) than into Cys (1.5‐fold); however, in all mutants, the opposite was true; the induction of incorporation in Cys was higher than into GSH (Figure 4D,F). In addition, a lower incorporation into Cys was observed in both *pyd4* lines under control conditions (Figure 4D). Collectively, these data demonstrate that MGL and PYD4 are important for proper induction of the flux through sulfate assimilation by sulfate starvation and for balancing of the sulfur fluxes between Cys and GSH. Additionally, PYD4 plays a crucial role for control of the sulfur fluxes in the roots.

### PYD4 regulates sulfate starvation transcriptional network

**Figure 5:**
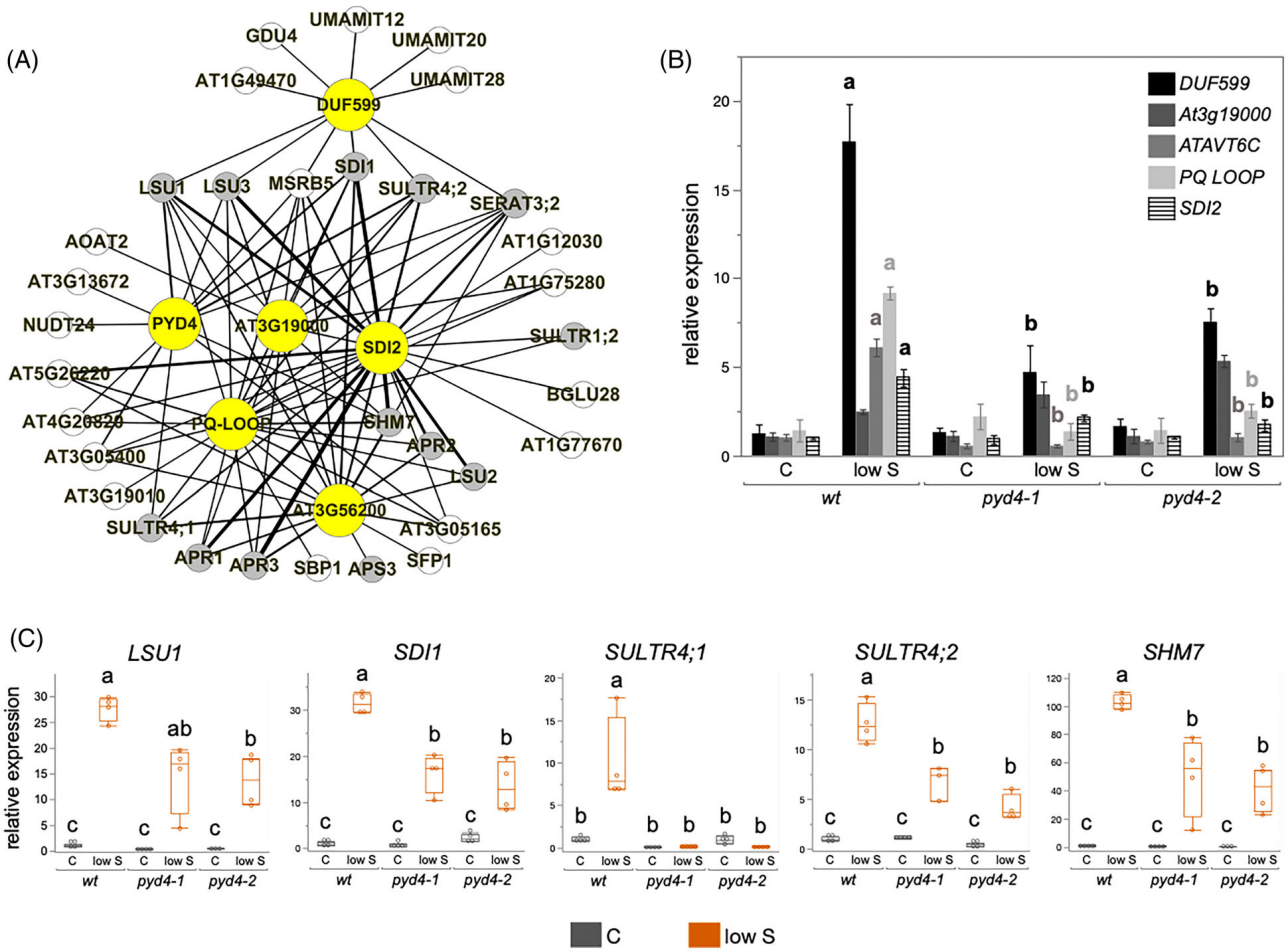
Sulfate transcriptional network is misregulated in *pyd4* mutants. (A) Sulfate transcriptional network is integrated among six of the seven shared genes in the four species. The six shared genes between the two monocots and two dicots, are depicted in yellow hubs. Co‐expression ranks were obtained from *atted.jp* for the seven genes (see Methods) and used for network construction using Cytoscape. Edge width corresponds to higher co‐expression rank. (B) Relative expression analysis of the five shared genes in the PYD4 network, in wild‐type (*WT*) and two independent mutant *pyd4* lines. (C) Relative expression analysis of sulfate marker genes directly connected to PYD4 in the co‐expressed network. Data are means ± SE; *n* = 4 biological replicates. Different letters indicate statistically significant difference for two‐way anova, and Tukey's HSD test for multiple comparisons. Plants were grown for 18 days on either 0.75 mM SO_4_^2−^ (C) or 0.015 mM SO_4_^2−^ (low S). Relative gene expression was determined via reverse transcription quantitative PCR (RT‐qPCR), normalized to two housekeeping genes in plant roots. *APRs, ADENOSINE‐5′‐PHOSPHOSULFATE REDUCTASEs; LSU, RESPONSE TO LOW SULFUR; PYD4, PYRIMIDINE 4; SDIs, SULFUR DEFICIENCY‐INDUCED; SHM7, serine hydroxymethyltransferase 7; SULTRs, SULFATE TRANSPORTERS; ATAVT6C, AT3G56200; PQ‐LOOP, AT5G40670; 2OG, AT3G19000; DUF599, AT4G31330*.

To examine the transcriptional connectivity of the seven shared S deficiency‐responsive genes, we used publicly available transcriptomic datasets (see Methods) to construct a co‐expression network (Figure 5A). Interestingly, five of the seven genes were strongly interconnected with each other and with the established sulfate starvation response network. In contrast, *DUF599* was only indirectly connected to the other five genes, while *MGL* formed a distinct cluster with only five edges (Figure S3). Notably, *ATL80*, a RING E3 ubiquitin ligase implicated in cold response, flower development, phosphate transport (Suh & Kim, 2015), and more recently in sulfate starvation regulation in monocots (Zenzen et al., 2024), was directly linked to *MGL*. Given these interconnections, we hypothesized that expression of the network genes might be perturbed in PYD4‐deficient lines. Indeed, transcript analysis revealed significant attenuation of *DUF599* and *SDI2* induction by sulfate starvation in two *pyd4* mutant lines, while *ATAVT6C* and *PQ‐LOOP* transcript levels under sulfate starvation were indistinguishable from those in control plants (Figure 5B). Within this network, *PYD4* itself was directly associated with canonical sulfate starvation marker genes, including *LSU1, LSU3, SDI1, SULTR4;1, SULTR4;2*, and *SHM7*, all previously validated as robustly induced under sulfur deficiency in *A. thaliana* (Dietzen et al., 2020; Maruyama‐Nakashita et al., 2006). To test whether PYD4 affects this core network, we measured transcript levels of sulfate transporters, assimilation enzymes, and regulatory genes under control and sulfate starvation conditions. Both *pyd4* mutant alleles exhibited markedly decreased expression of *LSU1, LSU2, LSU3, SDI1, SULTR4;1, SULTR4;2*, and *SHM7* under sulfate starvation (Figure 5C; Figure S3B). Collectively, these findings demonstrate that PYD4 is an integral component of the sulfate starvation transcriptional network, modulating sulfate transport and homeostasis regulatory pathways. PYD4 influences the expression of four of the seven novel conserved sulfate starvation‐responsive genes, underscoring its central role in coordinating plant adaptation to sulfur deprivation.

### PYD4 is required for photorespiration during sulfate starvation

**Figure 6:**
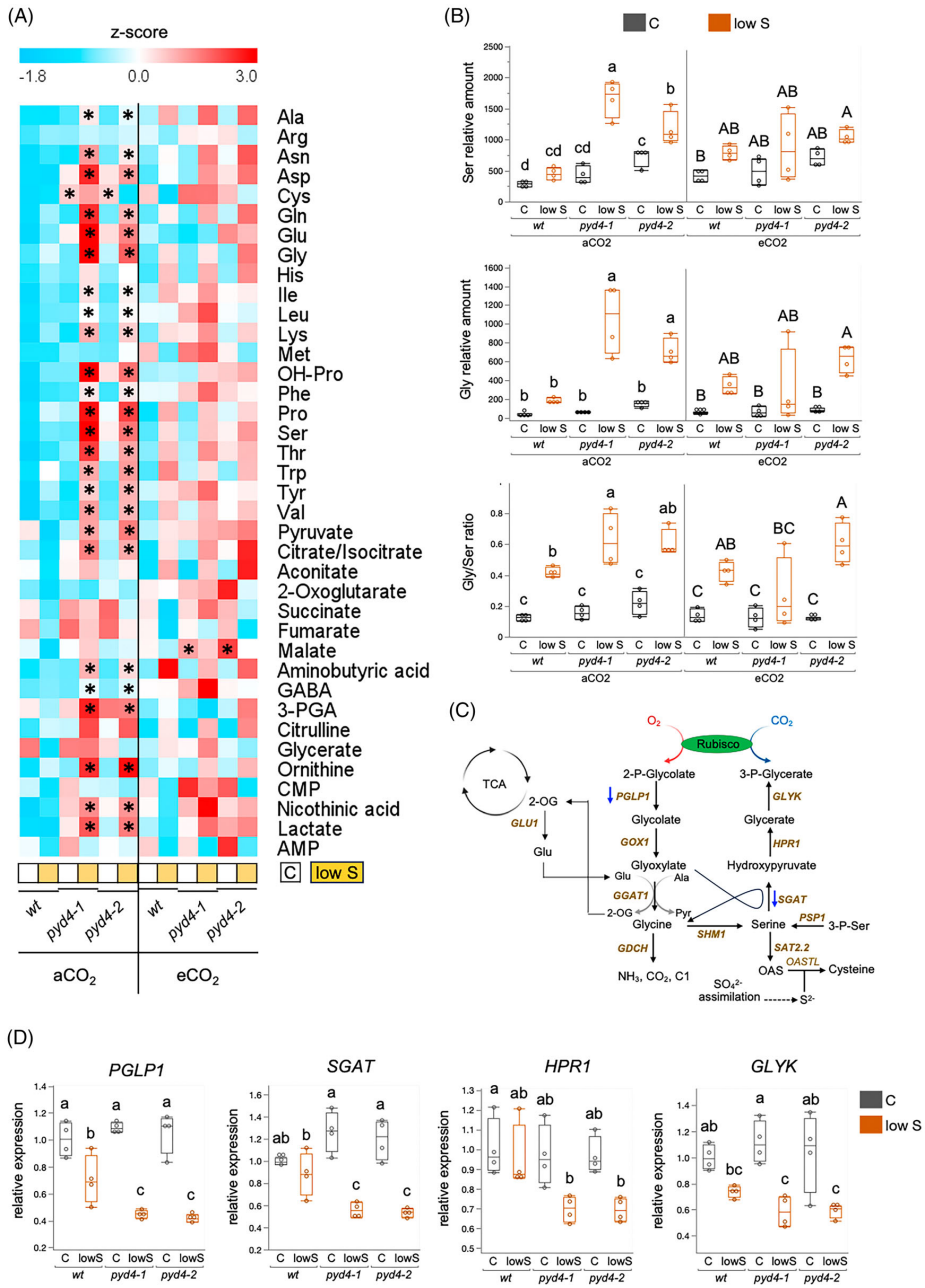
PYD4 function is required for photorespiration under S deficiency. (A) Heat map of metabolite profiling in two independent *pyd4* mutant lines under full media (C), and low sulfate media (low S), and ambient (aCO_2_) or elevated (eCO_2_) in shoots. *Z*‐scores of mean values from four biological replicates are shown. Asterisks indicate statistically different means when using two‐way anova, and Tukey's test for multiple comparisons (*n* = 4). (B) Relative amount of key photorespiration amino acids, serine (Ser), glycine (Gly) and their ratio in the same conditions as above. Data are means ± SE; *n* = 4 biological replicates. Different letters indicate statistically significant difference for two‐way anova, and Tukey's HSD test for multiple comparisons. Small letters refer to statistical test for aCO_2_, while capital letters refer to statistical test for eCO_2_. (C) Schematic representation of key photorespiration steps; the two significantly downregulated genes in two independent *pyd4* mutant lines are noted with blue down arrow. Genes depicted in bold letters were tested. (D) Relative gene expression of photorespiration genes in the shoots was determined via reverse transcription quantitative PCR (RT‐qPCR), normalized to two housekeeping genes. Data are means ± SE; *n* = 4 biological replicates. Different letters indicate statistically significant difference for two‐way anova, and Tukey's HSD test for multiple comparisons. Arabidopsis plants (15 seedlings) were grown vertically on agar plates for 18 days under full media (C, 0.75 mM SO_4_^2−^) and low sulfate media (low S 0.015 mM SO_4_^2−^), and ambient (aCO_2_, 400 ppm) or elevated (eCO_2_, 3000 ppm). Shoots from one plate (15 seedlings) were bulked into one biological sample, and metabolites or RNA extracted. Genotypes: Col‐0 (*wt*), *mgl‐1*, *mgl‐2*, *pyd4‐1*, *pyd4‐2*. *GLYK, glycerate kinase; HPR, hydroxypyruvate reductase 1; PGLP1, 2‐phosphoglycolate phosphatase 1; SGAT, l‐serine:glyoxylate aminotransferase*.

**Figure 7:**
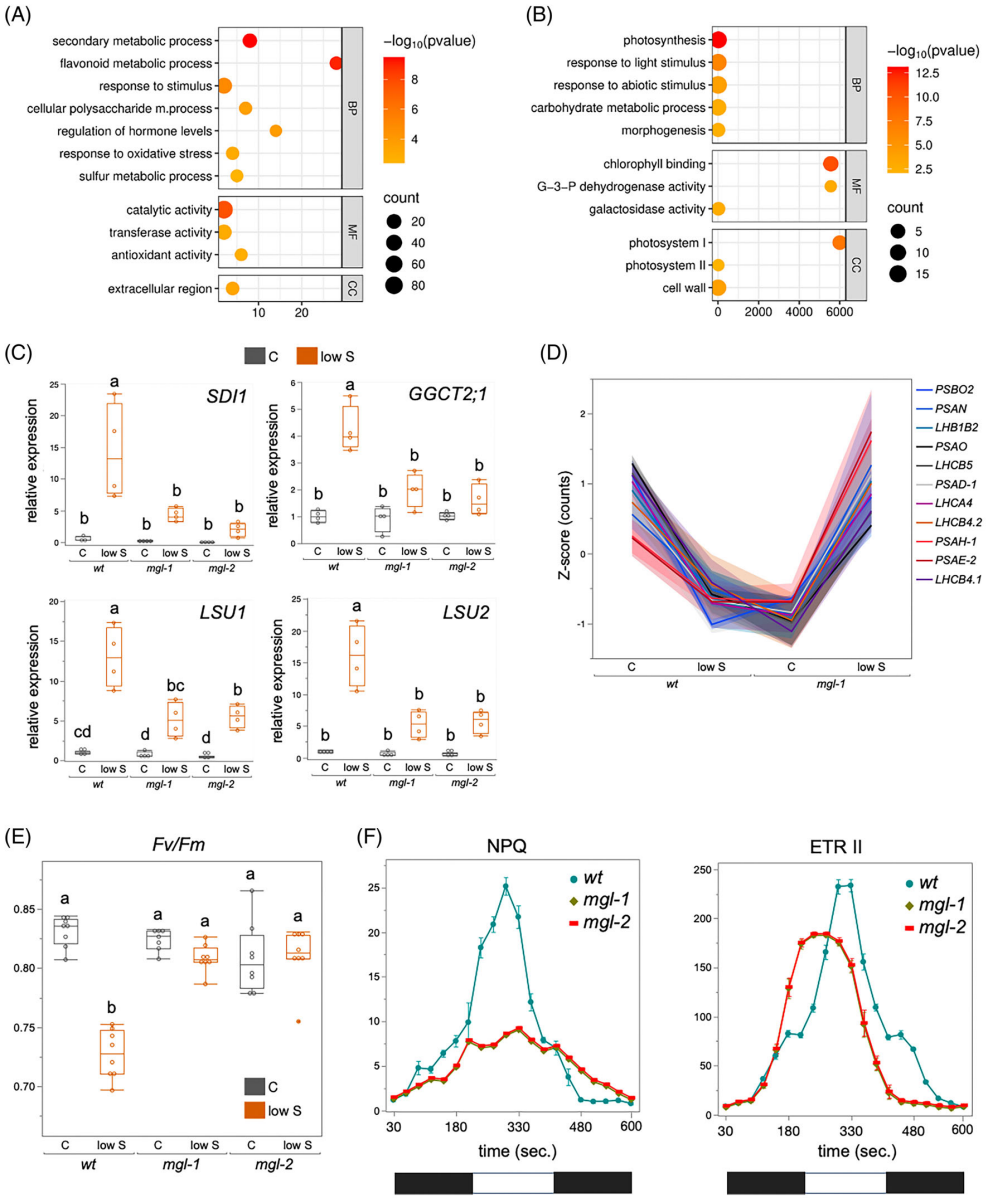
Transcriptomic profile of MGL‐deficient (*mgl*) mutant under S deficiency. (A) Selected gene ontology (GO) enrichment analysis on 176 downregulated differentially expressed genes (DEGs) responding to interaction effect. (B) Selected GO enrichment analysis on 159 upregulated DEGs responding to interaction effect. Biological GO terms (BP), molecular (MF), and cellular (CC). (C) Relative expression of sulfate starvation marker genes attenuated in both *mgl* mutants. Data are means ± SE; *n* = 4 biological replicates. Different letters indicate statistically significant difference for two‐way anova, and Tukey's HSD test for multiple comparisons. Relative gene expression was determined via reverse transcription quantitative PCR (RT‐qPCR), normalized to two housekeeping genes in plant roots. (D) *Z*‐scores of 11 genes coding for subunits of PSI and PSII and light‐harvesting complexes that were mis‐regulated in the *mgl‐1* mutant under S deficiency. (E) Impact of sulfate starvation and *MGL* mutation on the quantum efficiency of open photosystem II centers (*F*_v_/*F*_m_), and (F) non‐photochemical quenching (NPQ), and electron transport rate at PS II (rETR II). Data are means ± SE; *n* = 7–8 biological replicates. Different letters indicate statistically significant difference for two‐way anova, and Tukey's HSD test for multiple comparisons. Plants were grown for 18 days on either 0.75 mM SO_4_^2−^ (C) or 0.015 mM SO_4_^2−^ (low S).

*PYD4* belongs to the *PYD* gene family, with *PYD1‐3* primarily known for regulating the uracil degradation pathway (Zrenner et al., 2009). To explore whether *PYD4* influences pyrimidine metabolism, we examined the expression of six genes involved in *de novo* pyrimidine biosynthesis, phosphotransfer, and the nucleotide salvage pathway that were previously reported to respond to mutations in *PYD1‐3* (Zrenner et al., 2009). None of these genes were significantly altered in *pyd4* mutants (Figure S4), suggesting that *PYD4* has a distinct function beyond uracil catabolism. In fact, *PYD4* was shown to encode an l‐alanine:glyoxylate aminotransferase, while a β‐alanine:pyruvate aminotransferase activity was not detected (Parthasarathy et al., 2019), suggesting its involvement in photorespiration. Recent systematic characterization of Arabidopsis aminotransferases (AT) substrate specificity showed that three poorly characterized putative mitochondrial AT, including PYD4, exhibited alanine:hydroxypyruvate and alanine:glyoxylate AT activities. PYD4 had almost identical pattern as AGT3 (ALANINE:GLYOXYLATE AMINOTRANSFERASE 3), supporting the hypothesis that it might be involved in photorespiration (Koper et al., 2025). To test this hypothesis, we performed metabolic profiling of amino acids, organic acids, and other metabolites in plants grown on full media, and under sulfate starvation, both at ambient and elevated CO_2_. Remarkably, under ambient CO_2_, 25 metabolites, predominantly amino acids, accumulated significantly more in both *pyd4* mutant lines than in WT under sulfate starvation (Figure 6A). Of the 21 amino acids measured, 18 increased in the mutants under these conditions, with glycine (Gly) and serine (Ser) exhibiting the strongest elevations (Figure 7B). Ser levels increased 2–3‐fold, while Gly levels surged 3.5–6.5‐fold compared with WT, indicating a pronounced disruption of glycine metabolism relative to serine under sulfate starvation (Figure 6B).

To pinpoint where photorespiration is affected in the PYD4‐deficient mutants under sulfate starvation, we quantified expression of photorespiratory genes (Figure 6C,D; Figure S5). The entry step of the photorespiratory pathway, the conversion of 2‐phosphoglycolate (2PG) to glycolate, is catalyzed by 2PG phosphatases (PGLP1) in the plastids (Schwarte & Bauwe, 2007). In both PYD4‐deficient lines, *PGLP1* expression was significantly downregulated under sulfate starvation (Figure 6D). Similarly, the transcript for serine:glyoxylate aminotransferase (SGAT), which converts glyoxylate and serine to glycine and 3‐hydroxypyruvate (3‐HP) in the peroxisomes (Modde et al., 2017), was also downregulated in both *pyd4* lines (Figure 6D). Other photorespiratory genes showed a trend of decreased expression in the mutants under sulfate starvation (Figure 6D; Figure S5). In contrast, metabolic profiling of *mgl* and *eil3* mutants revealed only minor changes (Figure S6). Taken together, these results demonstrate that PYD4 is crucial for the integration of sulfate starvation responses with the photorespiratory pathway, particularly affecting glycine–serine metabolism and the expression of genes essential for photorespiratory flux. This disruption likely underlies the metabolic and physiological phenotypes observed in *pyd4* mutants during sulfate starvation.

### MGL regulates diverse processes under sulfate starvation including photosynthesis

Surprisingly, metabolite profiling of both *mgl* lines revealed only a single significant change: increased ornithine levels under sulfate starvation (Figure S6). Ornithine is an intermediate in arginine (Arg) biosynthesis (Kalamaki et al., 2009), yet Arg levels remained unaffected. To further elucidate the role of MGL in sulfur homeostasis, we performed genome‐wide transcriptomic analysis of roots from WT and *mgl‐1* plants grown under full and sulfur‐deficient conditions. We first compared upregulated and downregulated DEGs in response to sulfate starvation in the WT versus the *mgl* mutant. There were 314 DEGs upregulated in the WT and 157 DEGs in the *mgl* mutant, of which only 45 overlapped (Figure S7A). Similarly, 332 downregulated DEGs were found in the WT, while 214 in the *mgl* mutant, and 37 overlapping, suggesting that the response to sulfate starvation is mostly distinct in the two genotypes (Figure S7A). This was corroborated by comparison of the overrepresented gene ontology (GO) terms. Only 9 GO terms overlapped between the upregulated DEGs responsive to sulfate starvation in WT and *mgl* mutant, while 44 and 14 were specific, respectively (Table S1). On the other hand, the number of overlapping GO terms for the downregulated DEGs was higher than in each genotype, but still numerous GO terms were specific for WT or *mgl* mutant (Table S1). Interestingly, 39 DEGs had opposite responses to sulfate starvation in the WT and the *mgl* mutant (Figure S7A).

To examine the specific contribution of the *mgl* genotype under sulfate starvation, we performed second DESeq2 analysis, and identified DEGs responsive to the main effect of *mgl‐1* genotype versus the interaction effect of *mgl‐1* under sulfate starvation (*mgl‐1*×*low S*). Only 3 DEGs were significantly affected by the *mgl* mutation, while 335 DEGs were found in the mutant sulfate starvation interaction term (Figure S7B), strongly suggesting that MGL function is particularly important during S starvation. We next examined the 335 *mgl*×*low S* regulated DEGs using GO (Maere et al., 2005). Among them, 176 downregulated DEGs were enriched in 68 biological GO terms, and 159 upregulated DEGs in 91 GO terms (Figure 7B,C; Table S2). The top GO terms among the downregulated DEGs were ‘*secondary metabolic process*’ and ‘*flavonoid metabolic process*’, suggesting that MGL‐mediated Met recycling under sulfate starvation is crucial for synthesis of secondary metabolites. ‘*Sulfur metabolic process*’, also appeared among enriched terms, highlighting the role of MGL in regulating S homeostasis. Consistent with this, several sulfur starvation marker genes, including *SDI1* and *GGCT2;1* (*γ‐Glutamylcyclotransferase*), showed attenuated response to sulfur deficiency in the *mgl* background. Quantitative reverse transcriptase‐polymerase chain reaction (qRT‐PCR) further confirmed that induction of *SDI1*, *GGCT2;1*, *LSU1*, and *LSU2* was attenuated in both *mgl‐1* and *mgl‐2* mutants (Figure 7C). Taken together, these results demonstrate that MGL is required for proper induction of sulfate starvation responses and integrates sulfate starvation response with diverse metabolic pathways.

To gain further insights, we performed clustering analysis and identified 17 co‐regulated clusters (Figure S7C; Table S3). GO term enrichment analysis (Maere et al., 2005) revealed clusters with a particular function or ‘biomodules’ (Nero et al., 2009) in nine clusters (Table S4). For instance, Cluster 4 contained genes unresponsive to sulfate starvation in the WT but upregulated under full media and downregulated under sulfate starvation in the *mgl* mutant. Biomodules, such as ‘*catalytic activity*’, ‘*oxidoreductase activity*’, ‘*electron carrier activity*’, and others (Table S4), point to a significant role of MGL in maintaining redox homeostasis and energy metabolism under sulfate starvation. Cluster 8 genes, upregulated by sulfate starvation in the WT, were downregulated in the mutant, with enriched terms linked to ‘*glucosyltransferase activity*’, suggesting altered metabolite modification and signaling. Cluster 10 genes, upregulated under sulfate starvation in the WT and upregulated constitutively in the *mgl* mutant, were enriched in ‘*auxin:hydrogen symporter activity*’, represented by *PIN5*. PIN5 belongs to the family of PIN‐FORMED (PIN) proteins of auxin efflux carriers and is involved in conveying auxin from the cytoplasm into the ER lumen (Mravec et al., 2009). Cluster 15 exhibited opposite responses between genotypes with enrichment for ‘*chlorophyll binding*’, pin‐pointing the role of MGL in photosynthesis during sulfate starvation. Taken together, these results suggest that MGL function during sulfate starvation is not only critical for sulfate starvation response but also for a range of distinctive metabolic processes to maintain redox balance, signaling, metabolite modifications, and photosynthetic efficiency.

Among the upregulated GO terms photosynthesis‐related GO terms, such as ‘*photosynthesis*’, ‘*chlorophyll binding*’, ‘*photosystem I*’ and ‘*photosystem II*’ were consistently enriched, with the corresponding genes upregulated in *mgl‐1* under sulfate starvation (Figure 7D; Tables S1 and S2). To assess the functional consequences, we grew plants in pots under control and sulfate starvation conditions (Figure S8), and quantified chlorophyll fluorescence parameters (Figure S9), including non‐photochemical quenching (NPQ) that indicates the dissipation of the excess light energy as heat in the chloroplasts to avoid damage to the photosynthetic apparatus, relative electron transport rate in photosystem II (ETR II) that reflects the efficiency of the light reactions of photosynthesis, and the maximum quantum efficiency of PSII (*F*_v_/*F*_m_) that indicates the maximum efficiency of Photosystem II (PSII) photochemistry (Hickey et al., 2022). Under control conditions, photosynthetic performance was largely comparable among WT and the two *mgl* mutant alleles (*mgl‐1* and *mgl‐2*), with no major differences observed in *F*_v_ */F*_m_ or NPQ (Figure S10A,B). However, a modest reduction in electron transport was detected in the mutants, as both *mgl‐1* and *mgl‐2* exhibited lower ETR I kinetics during the actinic light phase compared with the WT (Figure S10C). In contrast, under sulfate starvation conditions, clear differences in photosynthetic responses emerged. The WT displayed a significant reduction in the maximum quantum efficiency of PSII (*F*_v_ */F*_m_), indicative of photoinhibition, whereas both *mgl* mutants maintained *F*_v_ */F*_m_ values similar to control conditions (Figure 7E). Consistently, NPQ was strongly induced in the WT under sulfate limitation but remained substantially lower in the *mgl* mutants, particularly during high‐light exposure. ETR II was reduced in both *mgl* lines and had shifted response, indicating sustained photosynthetic electron flow (Figure 7D). In addition, the slow kinetics curves with P700 redox state measurements were similar between the genotypes under control conditions, but under sulfate starvation for both *mgl* mutants elevated values were observed (Figure S10C,D), suggesting that the mutants maintain a more oxidized electron pool of PS I and/or enhanced Photosystem activity during illumination. Together, these results demonstrate that, unlike the WT, *mgl* mutants maintain PSII efficiency, and exhibit reduced dependence on photoprotective energy dissipation under sulfate deficiency.

## DISCUSSION

Sulfate starvation response is central to processes that maintain sulfate homeostasis, such as sulfate uptake, allocation, and storage. This transcriptional response has been well characterized in Arabidopsis (Dietzen et al., 2020; Maruyama‐Nakashita et al., 2003, 2006; Nikiforova et al., 2003), as well as in other plant species (Canales et al., 2020; Zenzen et al., 2024). However, the identification of novel regulators and components of sulfate starvation response remains limited. Our study demonstrates that only seven genes comprise a conserved transcriptional response shared between two dicots and two monocots, with only *SDI2* previously known to modulate sulfate homeostasis and GSLs synthesis (Aarabi et al., 2016). The small number of overlapping genes likely reflects the stringent criteria used to define DEGs, as well as incomplete gene annotations, particularly in monocots (Zenzen et al., 2024). Despite diverse transcriptional networks operating across species, sulfate starvation induces common metabolic responses such as decreased foliar sulfate, cysteine, and GSH levels (Canales et al., 2020; Dietzen et al., 2020; Zenzen et al., 2024), indicating a conserved physiological impact on sulfur homeostasis. Importantly, sulfur assimilation fluxes are closely linked with metabolic fluxes through photorespiration and photosynthesis (Abadie & Tcherkez, 2019), but whether sulfur metabolism feeds back to these pathways has been unclear until now. Our findings reveal that PYD4 and MGL connect sulfate starvation responses with photorespiration and photosynthesis, respectively, integrating sulfur metabolism and primary carbon fluxes.

Consistent with the limited number of conserved sulfate‐responsive genes identified here, previous comparative transcriptomic studies across species and nutrient conditions have revealed predominantly species‐specific responses. For example, only 58 DEGs were shared between rice and setaria under sulfate starvation (Zenzen et al., 2024). Similarly, analyses of phosphate limitation across four species showed that only about half of the top phosphate‐responsive genes in *A. thaliana* are conserved in grasses and legumes (Pant et al., 2025). A comparable pattern has been observed for nitrogen starvation, where only 73 orthologous genes are shared between rice and *A. thaliana* (Cai et al., 2012). Even within a single species, responses can vary substantially: among two rice cultivars exposed to nitrogen limitation, only a small fraction of responsive genes overlapped (Sinha et al., 2018). Broader comparative studies of oxidative and hormone‐induced stress responses in Arabidopsis, rice, and barley further revealed that orthologous genes can display divergent or even opposing regulation, underscoring the extent of species‐specific adaptation (Hartmann et al., 2022). For instance, responses to methyl viologen‐induced oxidative stress differed markedly among the three species, while hormone treatments resulted in even less overlap in gene expression, highlighting the diversity of regulatory networks controlling stress responses (Hartmann et al., 2022).

### PYD4 within the sulfate starvation network

Our results place PYD4 as an integral component of the sulfate starvation network, where it influences multiple aspects of sulfur homeostasis. *PYD4* transcripts are highly upregulated under sulfate starvation but normalize to control levels within 60 min of sulfate resupply (Figure 1C), indicating a dynamic and tightly controlled response. This induction is dependent on SLIM1/EIL3, with expression more significantly affected in shoots than roots (Figure 2), highlighting PYD4's importance particularly in shoot‐based sulfate starvation responses. Notably, several phenotypes observed in *pyd4* mutants, especially those related to sulfur metabolism, resemble those reported for *slim1‐1* or *eil3* mutants (Dietzen et al., 2020; Kawashima et al., 2011). For example, PYD4 contributes to the maintenance of sulfate, GSH, and aliphatic GSLs pools already at full media (Figure 3A,C,D), comparably to SLIM1/EIL3 (Dietzen et al., 2020; Kawashima et al., 2011). At the transcriptional level, *pyd4* mutants show an attenuated response to sulfur deficiency, comparable to *slim1‐1* and *eil3*, although generally less severe (Dietzen et al., 2020; Maruyama‐Nakashita et al., 2006). A notable exception is *SULTR4;1*, whose induction under sulfate limitation is completely abolished in *pyd4* mutants (Figure 5C). *SULTR4;1* and *SULTR4;2* encode tonoplast‐localized sulfate transporters involved in intracellular sulfate remobilization (Kataoka et al., 2004), and their transcripts were upregulated under sulfate starvation and attenuated in the *eil3* background (Dietzen et al., 2020). The strong effect on *SULTR4;1* suggests that PYD4 plays a key role in regulating vacuolar sulfate fluxes during sulfur starvation.

Beyond known marker genes, PYD4 also positively regulates four of the seven conserved sulfate starvation genes identified in this study (Figure 5B), further supporting its role in the sulfate starvation response. Interestingly, *DUF599*, *ATAVT6C*, and *PQ‐LOOP* were reported to be attenuated in the *eil3* background under S deficiency, but not *SDI2* (Dietzen et al., 2020), indicating overlapping yet distinct regulatory roles for SLIM1/EIL3 and PYD4. Although the expression patterns of sulfate starvation marker genes are similar between *slim1* or *eil3* and *pyd4* lines (Dietzen et al., 2020; Maruyama‐Nakashita et al., 2006), PYD4 itself is unlikely to act as a primary transcriptional regulator. Instead, its similar expression profile and downstream effects suggest that it provides an additional regulatory layer, potentially linking transcriptional control with metabolic adjustments required for maintaining sulfur homeostasis.

### Integration of sulfur flux and photorespiration

Previous work has linked PYD4 to stress responses and pyrimidine catabolism, as its expression is induced by osmotic stress and co‐regulated with uracil degradation genes (Zrenner et al., 2009). Although initially annotated as a β‐alanine aminotransferase, phylogenetic analyses placed PYD4 in a distinct clade (Wu et al., 2016), suggesting functional divergence. Recently, this view was reinforced by biochemical evidence showing that PYD4 exhibits alanine:hydroxypyruvate and alanine:glyoxylate aminotransferase activities (Koper et al., 2025), directly connecting it to central photorespiratory intermediates. Our metabolic data further support a tight link between PYD4, sulfur metabolism, and photorespiration. Under sulfate starvation at ambient CO_2_, *pyd4* mutants accumulate a broad range of amino acids, most prominently serine and glycine, key intermediates of the photorespiratory pathway (Figure 6A; Figure S6). In parallel, induction of *PGLP1* and *SGAT*, encoding plastidial and peroxisomal photorespiratory enzymes, is attenuated, indicating reduced photorespiratory flux. Given that photorespiration is essential for detoxifying 2PG and sustaining Calvin–Benson cycle activity (Timm et al., 2025), these findings point to a functional impairment of the pathway in the absence of PYD4. Importantly, photorespiratory serine (glycolate pathway of serine biosynthesis, GPSB) represents a major precursor for cysteine synthesis and thus directly links carbon flux to sulfur assimilation (Samuilov et al., 2018).

Genetic evidence highlights a close integration between serine biosynthesis and sulfur homeostasis. Disruption of the phosphorylated pathway of serine biosynthesis (PPSB), as observed in *psp1* or *pgdh1* mutants, triggers transcriptional sulfate starvation responses, including strong upregulation of *PYD4* (Anoman et al., 2019; Rosa‐Téllez et al., 2024). However, combining PPSB defects with impaired photorespiratory serine metabolism (e.g., *shmt1 pgdh1*) reverses this response, underscoring functional interplay between PPSB and the photorespiratory glycolate pathway (GPSB). This interaction is further supported by evidence that disrupted photorespiration perturbs sulfate assimilation and its coordination with carbon and nitrogen metabolism (Samuilov et al., 2018). Within this network, *PYD4* appears to act as a key regulatory node. Reduced expression of *PGLP1* and *SGAT* in *pyd4* mutants, along with increased serine and glycine accumulation (Figure 6), suggests diminished photorespiratory flux. Induction of *PYD4* under PPSB deficiency may therefore compensate by sustaining serine production via GPSB, supporting downstream cysteine synthesis and sulfur metabolism.

Two alternative scenarios may explain PYD4 function in this context. PYD4 could directly participate in photorespiratory reactions, modulating glyoxylate and hydroxypyruvate interconversions and thereby sustaining pathway flux. The observed metabolic phenotype resembles that of photorespiratory mutants (Dellero et al., 2021), supporting this possibility. Alternatively, PYD4 may act indirectly via sulfur metabolism or signaling, where impaired sulfur status in *pyd4* mutants constrains photorespiration through transcriptional and metabolic reprogramming (Kopriva et al., 2019). The suppression of the phenotype under elevated CO_2_ conditions that reduce photorespiratory demand supports this interpretation.

### MGL within the sulfate starvation network

Although *MGL* is not co‐expressed with the other six shared genes in the sulfate starvation network, our data showed the importance of MGL in recycling S pools during sulfate starvation and how its function impacts diverse processes in the plant. MGL, an enzyme that degrades Met to α‐ketobutyrate, methanethiol (CH_3_SH), and NH_3_, is encoded by a single copy gene in Arabidopsis, *O. sativa* Kitaake, and *S. viridis*, but by three genes in *S. lycopersicum* (Goodstein et al., 2012). Plants possess an alternative to the reverse trans‐sulfuration pathway, enabling synthesis of Cys from Met, in which MGL and methanethiol play a crucial role. Indeed, the *mgl* mutant fed with [^35^S]Met incorporated less ^35^S into Cys than WT, when plants were grown in a low sulfate medium (Goyer et al., 2007). Our metabolic data also suggested that Met recycling is important for homeostasis of other S‐metabolites, such as GSH and aliphatic GSLs, which are derived from Met, and were increased under sulfate starvation in the shoots of MGL‐deficient lines (Figure 3C,D). However, flux through sulfate assimilation pathway measured as [^35^S] incorporation from [^35^S]sulfate into Cys and GSH in the leaf was unchanged, but strongly attenuated in the roots for Cys, while incorporation into GSH was increased in the *mgl* mutants under sulfate starvation (Figure 4). Early studies showed that plants fed with L‐Met or S‐methylcysteine (SMC) emitted volatile methanethiol. However, methanethiol produced in intact leaves was not released into the atmosphere as hydrogen sulfide is, suggesting that methanethiol is retained and recycled in the leaf, and is only released upon damage to the veins (Schmidt et al., 1985). Moreover, methanethiol might act as a signal for sulfate uptake, being derived from the shoots and transported to the roots (Schmidt et al., 1985). Thus, consistent with this hypothesis, our data indicate that MGL is essential for proper Met recycling, regulation of sulfur metabolites, and their balance between roots and shoots during sulfur deficiency.

MGL dysfunction could affect diverse processes in the plant, as we observed in this study. Interestingly, the genotype effect of the MGL‐deficient line had little consequence at the transcriptional level (Figure S7A). However, the interaction effect of the MGL‐deficient genotype and sulfur deficiency had a substantial impact on diverse processes. The ‘*sulfur metabolic processes*’ GO term was enriched among the downregulated DEGs, and inductions of key sulfur starvation sentinel genes were attenuated in both *mgl* mutant lines (Figure 7C,E). *MGL* expression is upregulated by osmotic and drought stress, which might be associated with another catabolic product of MGL, α‐ketobutyrate, being a substrate for isoleucine (Ile) synthesis. Ile plays an important role under stress by acting as an osmoprotectant, and *mgl* mutants incorporated significantly less radioactive carbon from Met into Ile under drought stress (Joshi & Jander, 2009). Our transcriptomics data identified few stress‐related GO terms, such as ‘*osmotic*’, ‘*oxidative*’, and ‘*salt*’ overrepresented in the *mgl* mutant under sulfate starvation (Table S1), indicating the important role of MGL not only in recycling sulfur compounds, but also other metabolites, and thus coordinating S homeostasis with stress responses.

### Significance of MGL in photosynthetic efficiency

The combined physiological, metabolic, and transcriptomic data reveal a central role for MGL in coordinating sulfur metabolism with photosynthetic performance and oxidative stress responses under sulfate starvation. Early evidence already suggested a link between MGL activity and light‐dependent processes, as feeding plants with l‐methionine induced the emission of volatile methanethiol in both darkness and light, with enhanced emission under illumination (Schmidt et al., 1985). Consistent with this connection, our transcriptomic analysis showed that sulfate starvation in the *mgl‐1* mutant leads to the upregulation of multiple genes associated with the light reactions of photosynthesis, including subunits of PSI, PSII, and light‐harvesting complexes (Figure 7D). This transcriptional reprogramming suggests an active adjustment of the photosynthetic machinery in response to disrupted methionine catabolism.

At the physiological level, WT plants displayed a classical stress response to sulfate starvation (Lunde et al., 2008), consistent with responses observed under other macronutrient deficiencies such as phosphorus and nitrogen (Calzadilla, 2024), characterized by increased NPQ and a decline in the maximum quantum efficiency of PSII (*F*_v_ */F*_m_), indicative of photoinhibition (Figure 7E,F). The parallel increase in NPQ and decrease in *F*_v_ */F*_m_ suggests that NPQ is predominantly driven by the photoinhibitory component (qI), rather than the rapidly reversible qE component (energy‐dependent photoprotective component), indicating insufficient photoprotection (Zuo, 2025). In contrast, both *mgl* mutant alleles showed little to no change in *F*_v_ */F*_m_ under sulfate starvation and exhibited reduced NPQ and electron transport rates, indicating a markedly attenuated photoinhibitory response. Under control conditions (Figure S9), only minor differences were observed, with slightly lower ETR I kinetics in the mutants during actinic illumination, suggesting that the protective phenotype is specific to stress conditions rather than a constitutive enhancement of photosynthetic capacity.

The apparent discrepancy between enhanced expression of photosynthetic genes and reduced NPQ and ETR kinetics in the *mgl* mutant suggests the activation of compensatory mechanisms that balance energy capture with redox homeostasis. Previous observations also suggest that the increased transcript levels of photosynthesis genes under abiotic stress are a part of an acclimatory response (Anwar et al., 2024; de Silva et al., 2025; Herrmann et al., 2021). Upregulation of PSI/PSII components and light‐harvesting complexes may serve to maintain efficient energy transfer and electron flow under conditions where metabolic constraints would otherwise limit photosynthetic output. At the same time, improved redox buffering reduces the need for energy dissipation via NPQ, resulting in a phenotype characterized by stable PSII efficiency and lower photoprotective demand.

Together, these findings support a model in which MGL‐mediated methionine catabolism plays a critical role in regulating sulfur economy under sulfate starvation, influencing the balance between metabolic sulfur use and redox protection. Loss of MGL activity shifts this balance toward enhanced GSH‐dependent antioxidant capacity, thereby mitigating oxidative stress and preserving photosynthetic function under sulfate starvation. This highlights methionine recycling as a key integrator of nutrient signaling, redox homeostasis, and photosynthetic performance in plants.

## CONCLUSIONS

While our comparative transcriptomic analysis identified a conserved sulfate starvation response across multiple species, functional validation in this study is limited to *A. thaliana*. Although the conserved transcriptional signatures point to shared regulatory components, they do not necessarily imply identical biochemical or physiological roles across diverse plant lineages. Future studies will be required to directly test whether the functions of PYD4 and MGL are conserved beyond Arabidopsis. An additional consideration is that our comparative dataset includes both wild and domesticated species. Domestication has shaped plant metabolic and regulatory networks through selection for yield, growth, and environmental adaptation, which can alter nutrient signaling pathways, including those related to sulfur metabolism. Therefore, the extent to which sulfate starvation responses and their integration with primary metabolism are conserved may vary between species, particularly in crops that have undergone intensive breeding. Despite these limitations, the identification of a small set of conserved sulfate‐responsive genes, including PYD4 and MGL, suggests the existence of a core regulatory module linking sulfur homeostasis with primary metabolism. In Arabidopsis, our data support roles for PYD4 and MGL in connecting sulfate availability with photorespiration and photosynthesis, respectively. It is plausible that orthologs of these genes in crop species contribute to similar processes, particularly in optimizing nutrient use efficiency under sulfate starvation conditions. However, their precise functions may have diverged or been rewired during domestication.

Understanding the interaction between mineral nutrition and other metabolic processes is vital for enabling crops to withstand environmental changes. Photorespiration and photosynthesis are common targets for crop improvement, and future breeding strategies must consider variation in abiotic factors such as atmospheric gaseous composition (CO_2_/O_2_ ratio) and nutrient availability. Additionally, nutrient use efficiency declines under unfavorable conditions. The metabolic interactions we describe here could have significant impacts on crop physiology and productivity. Our results provide biochemical and genetic evidence that the newly identified regulators of the sulfate starvation response, PYD4 and MGL, link sulfur metabolism with photorespiration and photosynthesis. Although, further investigations are needed to decipher the detailed mechanism how PYD4 and MGL affect photorespiration and photosynthesis under sulfate limitation. Since the transcriptional response of *PYD4* and *MG*L to sulfate starvation is conserved in two monocots and two dicots, these findings can help understand the multifaceted interactions of sulfur metabolism, and whether S‐assimilation should be studied in detail when photorespiration and photosynthesis are targeted by molecular engineering (Abadie & Tcherkez, 2019).

## MATERIALS AND METHODS

### Plant materials, growth conditions, and phenotypic analyses

*Arabidopsis thaliana* (Arabidopsis) seeds of WT (Col‐0 accession) and the five mutants were used in this study: *mgl‐1* (*SALK_040380*), *mgl‐2* (*SALK_103805*), *pyd4‐1* (*SAIL_400_G02*), *pyd4‐2* (*SALK_002102*) and previously published *eil3* line (Dietzen et al., 2020). Seeds were surface sterilized with chlorine gas using 125 ml NaOCl and 2.5 ml HCl (37%) for 3 h, after which sterile H_2_O was added for germination. The seeds were placed onto 0.8% agarose plates containing a modified Long‐Ashton medium or without agarose for the hydroponic set up (e.g., sulfate uptake) (Dietzen et al., 2020). The media consisted of 1.5 mM Ca(NO_3_)_2_ × 4H_2_O, 1 mM KNO_3_, 0.75 mM KH_2_PO_4_, 0.75 mM MgSO_4_ × 7H_2_O, and 0.1 mM Fe‐EDTA in terms of macroelements. Microelements consisted of 10 μM MnCl_2_ × 4H_2_O, 50 μM H_3_BO_3_, 1.75 μM ZnCl_2_, 0.5 μM CuCl_2_, 0.8 μM Na_2_MoO_4_, 1 μM KI, and 0.1 μM CoCl_2_ × 6H_2_O. In the low sulfate media, the 0.75 mM MgSO_4_ × 7H_2_O was replaced with a mixture of 0.7125 mM MgCl_2_ × 6H_2_O and 0.0225 mM MgSO_4_ × 7H_2_O, supplemented with 0.8 g L^−1^ MES salts, and 0.5% sucrose, and pH adjusted to 5.7 with KOH. The plates were placed at 4°C for 2 days for optimum germination and then incubated vertically for 18 days in Sanyo light chambers with a photoperiod consisting of a 16:8‐h light and dark cycle, with humidity at 60% and 21°C. Alternatively, for determination of sulfate uptake the plants were grown in 12‐well plates as described in Koprivova et al. (2023). Sterile seeds were distributed onto square sterile nylon membranes and placed in 12‐well plates on top of 1 ml of the modified Long‐Ashton medium with 0.5% sucrose. After stratification for 2 days in dark and cold, the plates were incubated for 3 days in dark and in 22°C to promote etiolation, and further for 2 weeks in the growth cabinets under the long‐day conditions as described above.

### Flux through sulfate assimilation

Flux through sulfate assimilation was determined in seedlings grown for 2 weeks in 12‐well plates on a modified Long‐Ashton medium under S‐sufficient or S‐deficient conditions. For the flux measurement the medium was exchanged with 1 ml of the Long‐Ashton medium with 0.2 mM sulfate supplemented with 0.1 μCi of [^35^S] sulfuric acid and incubated for 4 h in the light. Whole seedlings still on the mesh were washed thoroughly, blotted dry, and shoot and root samples were cut, weighed, and stored separately in liquid nitrogen until further processing on the same day. Samples were extracted in a 10‐fold volume of 0.1 m HCl. Ten microliters of extract were used to determine sulfate uptake, and 50 μl aliquots of each extract were collected for quantification of [^35^S] incorporation into thiols, exactly as in Mugford et al. (2011).

### RNA isolation and expression analysis

Total RNA was extracted from the roots of 18‐day‐old plants by standard phenol/chloroform extraction and LiCl precipitation. Thereafter, DNase treatment was performed and cDNA was synthesized from 600 ng of total RNA using the QuantiTect Reverse Transcription kit (QIAGEN, Germantown, MD, USA) according to the manufacturer's protocol. The product was diluted with autoclaved water to a final volume of 200 μl. Using quantitative real‐time PCR (qPCR), 11 genes were examined using gene‐specific primers indicated in Table S5. The qPCR was performed using the goTaq® qPCR Master Mix kit along with SYBR green as per the manufacturer's instructions using a CFX96 Touch Real‐Time PCR Detection System (Bio‐Rad). All quantifications were normalized to the two housekeeping genes (*ACT* and *CLA*) using the 2^−ΔΔCt^ method (Rosas et al., 2013). The RT‐PCR reactions were performed in duplicate for each of the four independent samples. All primers are listed in Table S4.

### Library preparation, RNA sequencing, and data processing and analysis

Quality control of RNA samples was performed before sequencing using the Bioanalyzer system (2100 Bioanalyzer Instrument, Agilent Technologies, Santa Clara, CA, USA), and the RNA 6000 Nano Kit (Agilent Technologies, Santa Clara, CA, USA), adopting a RIN cutoff value of at least 6 (2 μg; 50–200 ng μl^−1^; OD_260/280_ = 1.8–2.1; OD_260/230_ >1.5). The library preparation and RNA sequencing were performed by Novogene (Novogene Co., Ltd., Cambridge, UK). Libraries were sequenced using Illumina NovaSeq™ 6000 Sequencing System (Illumina, Inc., San Diego, CA, USA), to produce paired‐end 150 bp reads and coverage of 30–40 Mio reads per sample. Output data containing the paired‐end sequence data of each sample and their corresponding associated FastQC quality reports were stored as FastQ (fq.gz) files. HISAT2 version 2.1.0 (Kim et al., 2019) was used to align the sequenced fragments against the reference genome datasets from *A. thaliana* (‘TAIR11’) FASTA files, both obtained from the Joint Genome Institute via Phytozome portal (Goodstein et al., 2012). Read summarization was performed with *htseq‐count* (Anders et al., 2015), and the resulting count matrix was used for downstream analysis. Differential expression analysis was performed with DESeq2 (Love et al., 2014), where last replicate of mgl‐1 under sulfate starvation was removed from further analysis, due to lack of reduced *MGL* expression. Here, we used log2 fold change >1 or log2 fold change <−1, adjusted *P*‐value <0.05 cut off to obtain the DEGs. *Z*‐score was computed on a gene‐by‐gene basis by subtracting the mean and then dividing by the standard deviation. Clustering was performed using the JMP software, academic free version (https://www.jmp.com/en/academic). GO enrichment analysis was performed in BINGO app (Maere et al., 2005) in Cytoscape (Shannon et al., 2003).

### Sulfur‐related metabolite analysis

#### Anions

For the measurement of phosphate, nitrate and sulfate anions, 1 ml of sterile H_2_O was added to approximately 20 mg of homogenized shoot material shaken for 1 h at 4°C and heated to 95°C for 15 min. The samples were centrifuged at maximum speed for 15 min at 4°C, and 200 μl of the supernatants were transferred to an ion chromatography vial. Standard curves were generated using 0.5, 1, and 2 mM KH_2_PO_4_, KNO_3_, and K_2_SO_4_. The inorganic anions were measured with the Dionex ICS‐1100 chromatography system and separated using a Dionex IonPac AS22 RFIC 43250 mm analytic column (Thermo Scientific). The running buffer was made up of 4.5 mM NaCO_3_ and 1.4 mM NaHCO_3_ as described in Dietzen et al. (2020).

#### Thiols (Cys and GSH)

To analyze low molecular weight thiols (cysteine and GSH), approximately 20 mg of homogenized plant material was extracted with 0.1 m HCl at a 1:10 ratio (w/V) and subsequently centrifuged at maximum speed at 4°C. To reduce the thiols in the samples, 60 μl of the supernatant was transferred to a new tube and 100 μl 0.25 m CHES‐NaOH (pH 9.4) was added. Thereafter, 35 μl 10 mM dithiothreitol was added, the tubes were vortexed and incubated for 40 min at room temperature (RT). Five microliters of 25 mM monobromobimane was added to the reduced extracts, the samples were vortexed and incubated in darkness for 15 min at RT. The reaction was stopped by adding 110 μl of 100 mM methansulfonic acid and vortexing. After centrifugation at 4°C for 20 min, 200 μl of supernatant was transferred into high‐performance liquid chromatography (HPLC) vials. Standards, ranging from 0 to 100 mM, were prepared using 2 mM l‐cysteine and GSH stocks. The conjugated thiols were resolved using reverse phase (RP)‐HPLC (Eurospher 100‐3 C18, 150 × 4 mm; Knauer) and a gradient of 90% [v/v] methanol and 0.25% [v/v] acetic acid, pH 4.1 in 10% [v/v] methanol and 0.25% [v/v] acetic acid, pH 4.1, and detected fluorimetrically with a 474 detector with an excitation wavelength at 380 nm and emission wavelength at 470 nm. The flow rate was constant at 1 ml min^−1^.

#### GSLs (glucosinolates)

GSLs were extracted from approximately 20 mg homogenized plant material using 500 ml hot 70% (v/v) methanol, and 10 μl of sinigrin was added as internal standard. The extract was vortexed and incubated at 70°C for 45 min, vortexing twice during the incubation. The samples were left to cool and centrifuged at maximum speed for 5 min at RT. The supernatant was transferred to prepared columns containing 0.5 ml DEAE Sephadex A‐25, washed twice with 0.5 ml sterile H_2_O, and subsequently twice again with 0.5 ml of 0.02 m sodium acetate buffer. With a new tube placed underneath each column, a layer of 75 μl of sulfatase was placed onto the column. The samples were left at RT overnight, and the resulting desulfo‐GSLs were eluted twice with 0.5 ml sterile H_2_O, followed by a final elution of 0.25 ml. The eluates were combined, vortexed, centrifuged for 5 min, and 200 μl of the supernatant was transferred to HPLC vials. The desulfo‐GSLs were resolved by HPLC (Spherisorb ODS2, 250 3 4.6 mm, 5 μm; Waters) using a gradient of acetonitrile in water and detected by UV absorption at 229 nm. The GSLs were quantified using the internal standard and response factors as described in Dietzen et al. (2020).

#### Metabolic analysis (amino acids, organic acids, sugars)

Plant cultivation was on modified Long‐Ashton medium with agarose as described above, full media or S‐deficient media (Dietzen et al., 2020). Cultivation was carried out in controlled environment chambers (SANYO; 12/12‐h day/night cycle, 22°C/10°C, ~120 μmol m^−2^ sec^−1^ irradiance, 410 [aCO_2_] or 3000 [eCO_2_] ppm CO_2_, respectively). Whole plant rosettes were harvested at growth stage 1.04 (Boyes et al., 2001) in the middle of the light period (6 h illumination), frozen in liquid nitrogen and stored at −80°C. Prior to metabolite extraction, plant material was freeze‐dried by lyophilization.

Absolute abundances of selected primary metabolites were quantified through liquid chromatography coupled to tandem mass spectrometry (LC–MS/MS), using ~2–3 mg of dried plant weight (concentrations given in nmol mg DW^−1^). Extraction and LC–MS/MS measurements were carried out using LC–MS grade chemicals as described previously (Arrivault et al., 2009, 2015) and the modifications specified in Reinholdt et al. (2019). We used the ultra‐high‐performance liquid chromatograph mass spectrometer LCMS‐8050 system (Shimadzu) for metabolite measurements.

### Measurements of photosynthesis parameters using PAM (pulse amplitude modulation)

The plants were germinated and grown in 90% sand and 10% soil for 5 weeks under standard long‐day conditions. They were watered with VE‐water for the first 2 weeks, and after that with the liquid modified Long‐Ashton media (with or without SO_4_^2−^) twice per week. On the day of measurement, the plants were incubated in the dark for 15–20 min, and the seventh leaf was chosen from every plant for the PAM measurements. PAM measurements of chlorophyll fluorescence and P700 redox state were performed to assess photosynthetic parameters. *F*_v_ */F*_m_ and NPQ kinetics were recorded using a Dual‐PAM‐100 modular fluorimeter (Heinz Walz GmbH, Effeltrich, Germany). The red measuring light was set to 3 μmol photons m^−2^ sec^−1^, and saturating pulses to 4000 μmol photons m^−2^ sec^−1^ with a duration of 100 ms. Light response curves and NPQ were obtained using a chlorophyll fluorescence unit combined with a dual‐wavelength P700 detection system (830/875 nm), following Klughammer et al. (2013). Measurements were conducted across a range of light intensities: 21, 30, 43, 93, 164, 218, 372, 584, 909, and 1401 μmol photons m^−2^ sec^−1^. For slow kinetics, fluorescence curve measurements were obtained according to Steinberger et al. (2015).

### Statistical analysis

Statistical analysis was performed in JMP academic software (https://www.jmp.com/en/academic). To construct the sulfate starvation network, rank information for co‐expression of the seven shared genes was downloaded from https://atted.jp, and only *z*‐score ranks higher or equal than 5 were considered. The ranks were then scaled between 0 and 1, and co‐expression network was visualized in Cytoscape (https://cytoscape.org). Heat‐maps and clustering were created using mean *z*‐scores of raw values in MeV or JMP software (http://mev.tm4.org; https://www.jmp.com/en/academic). Enrichment bubble plots were created in SRplot (Tang et al., 2023).

## AUTHOR CONTRIBUTIONS

SB: methodology, validation, formal analysis, investigation, writing—review and editing; IT: methodology, investigation; ST: methodology, investigation, writing—review and editing; VS: methodology, formal analysis; PW: methodology, formal analysis; PB: methodology, formal analysis; SK: resources, supervision, project administration, funding acquisition, writing—review and editing; DR: conceptualization, methodology, validation, formal analysis, investigation, funding acquisition, project administration, resources, data curation, visualization, writing—original draft, writing—review and editing.

## CONFLICT OF INTEREST

The authors declare no competing interests.

## MATERIALS AND CORRESPONDENCE

The author responsible for distribution of materials integral to the findings presented in this article in accordance with the policy described in the Instructions for Authors (https://academic.oup.com/plcell/pages/General‐Instructions) is: Daniela Ristova (dristova@uni-koeln.de).

## Supporting information


**Table S1.** Intersect of GO‐enriched terms of upregulated and downregulated DEGs in WT versus *mgl‐1* under sulfate starvation.
**Table S2.** GO‐enriched terms in 335 DEGs regulated in *mgl‐1* under sulfate starvation.
**Table S3.** Hierarchical clustering of 335 DEGs regulated by *mgl*×*lowS*.
**Table S4.** Biological GO‐enriched terms in the 335 DEGs regulated in *mgl‐1* under sulfate starvation by cluster.
**Table S5.** Primers used in the study.
**Table S6.** Counts for RNA‐Seq analysis of *mgl‐1*.

**Figure S1.** Confirmation of T‐DNA insertion lines for PYD4 and MGL.

**Figure S2.** Anions and indolic GSLs quantification in shoot of two independent mutant lines of MGL and PYD4.

**Figure S3.** MGL co‐expression network and relative expression of sulfate starvation marker genes in two *pyd4* mutants.

**Figure S4.** Expression of pyrimidine nucleotide metabolism genes are not affected in *pyd4* mutant lines.

**Figure S5.** Expression of photorespiration genes that are not affected in two *pyd4* mutant lines.

**Figure S6.** Heat map of metabolite profiling in *mgl‐1, mgl‐2, pyd4‐1, pyd4‐2*, and *eil3* mutant lines under full media (C), and low sulfate media (low S), and ambient (aCO_2_) or elevated (eCO_2_) in shoots.

**Figure S7.** Transcriptome analysis of *mgl‐1* mutant.

**Figure S8.** Morphological and biochemical phenotypes of greenhouse grown plants for photosynthetic traits.

**Figure S9.** Impact of S deficiency on the photosynthetic parameters.

**Figure S10.** Impact of sulfate starvation on the photosynthetic parameters.

## Data Availability

RNA‐Seq counts generated in this manuscript are available in the Supporting Information (Table S6).
