# Supplementary figures and images for: Sulfate starvation response modules connect sulfur metabolism to photorespiration and photosynthesis
Source: Plant J. 2026 Jul 9;127(1):e71019. doi: 10.1111/tpj.71019 (PMC13349389; doi:10.1111/tpj.71019)

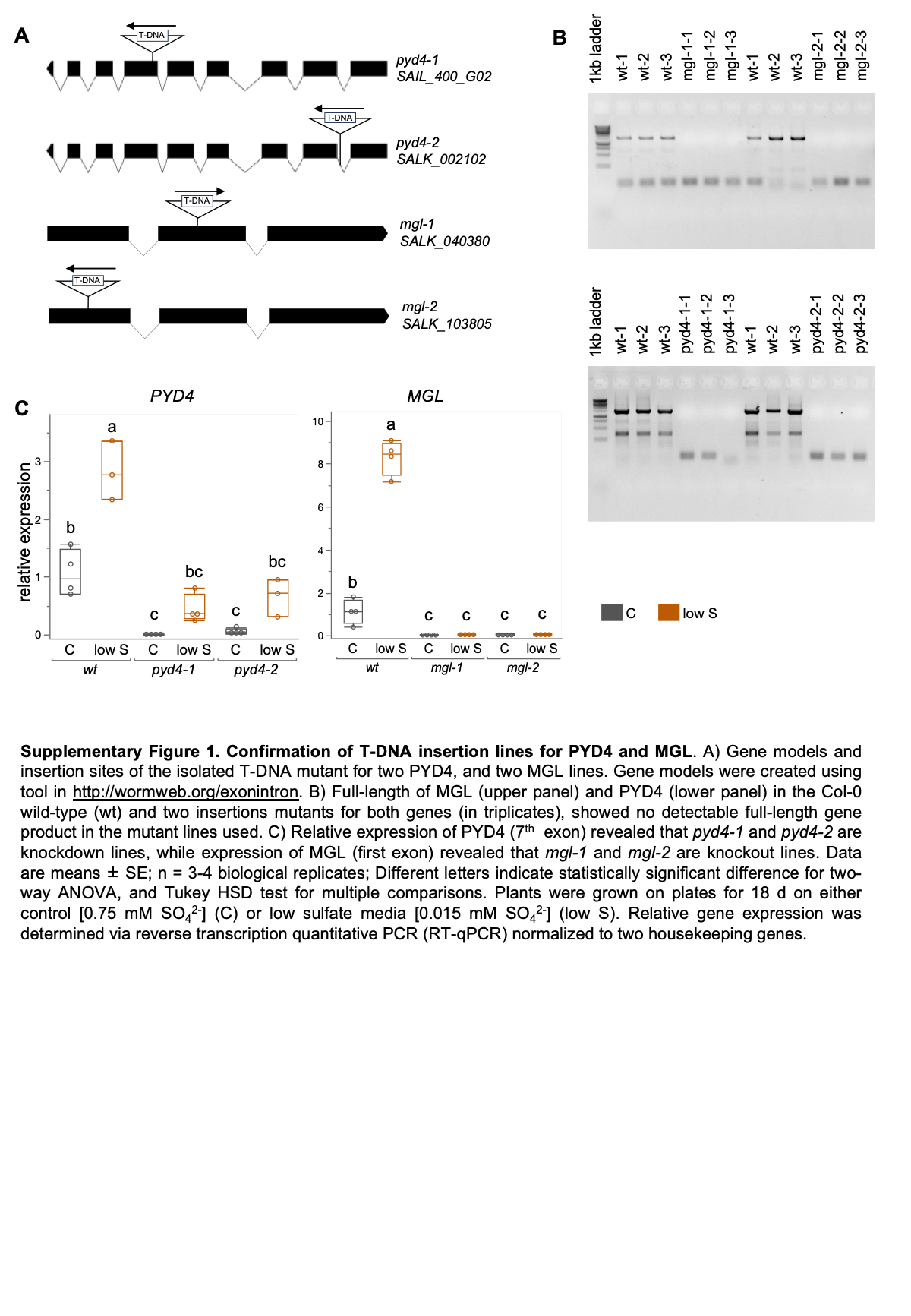

Supplement: Supplementary file 2 — Figure S1. Confirmation of T‐DNA insertion lines for PYD4 and MGL. [file TPJ-127-0-s001.png]

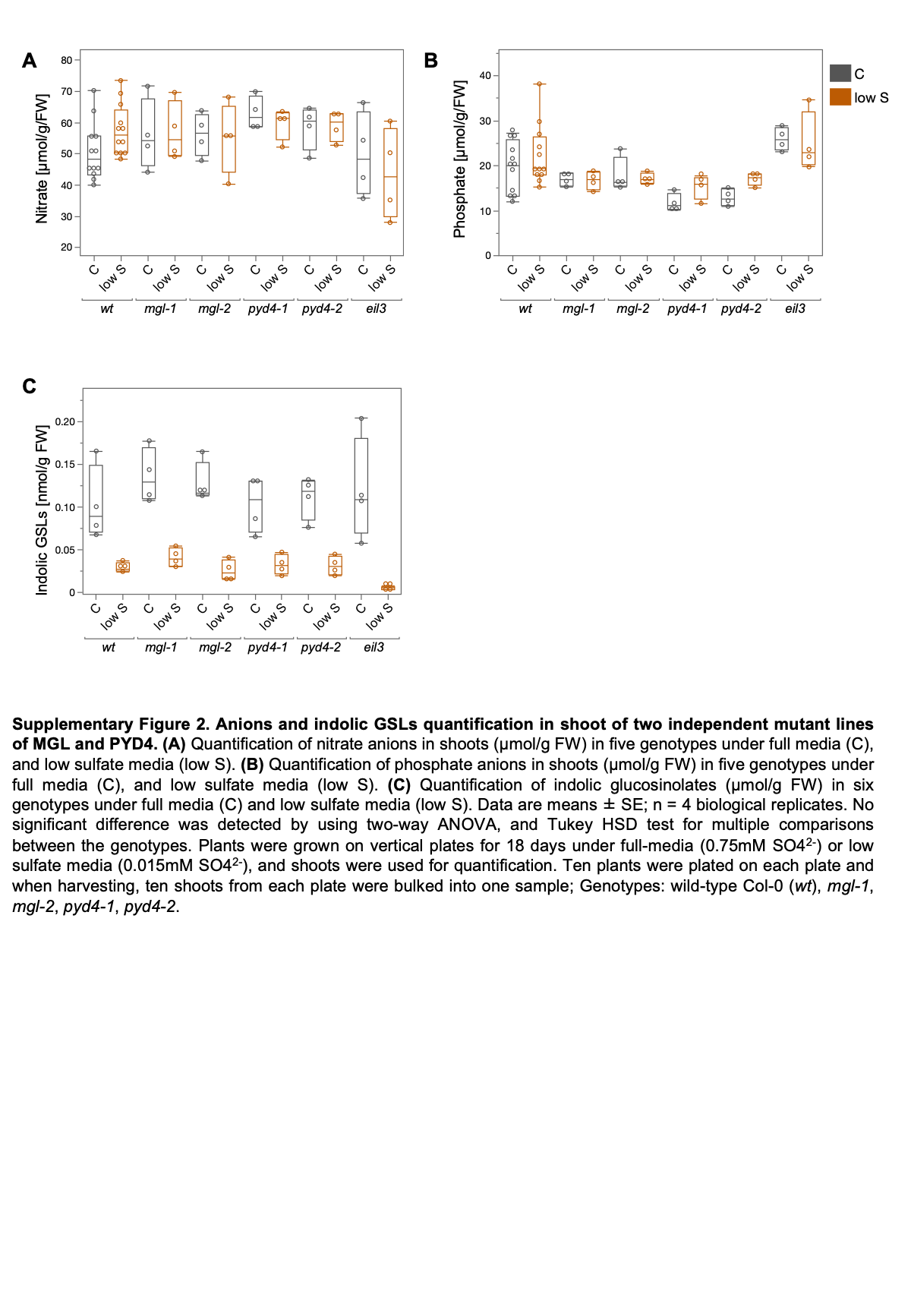

Supplement: Supplementary file 3 — Figure S2. Anions and indolic GSLs quantification in shoot of two independent mutant lines of MGL and PYD4. [file TPJ-127-0-s004.png]

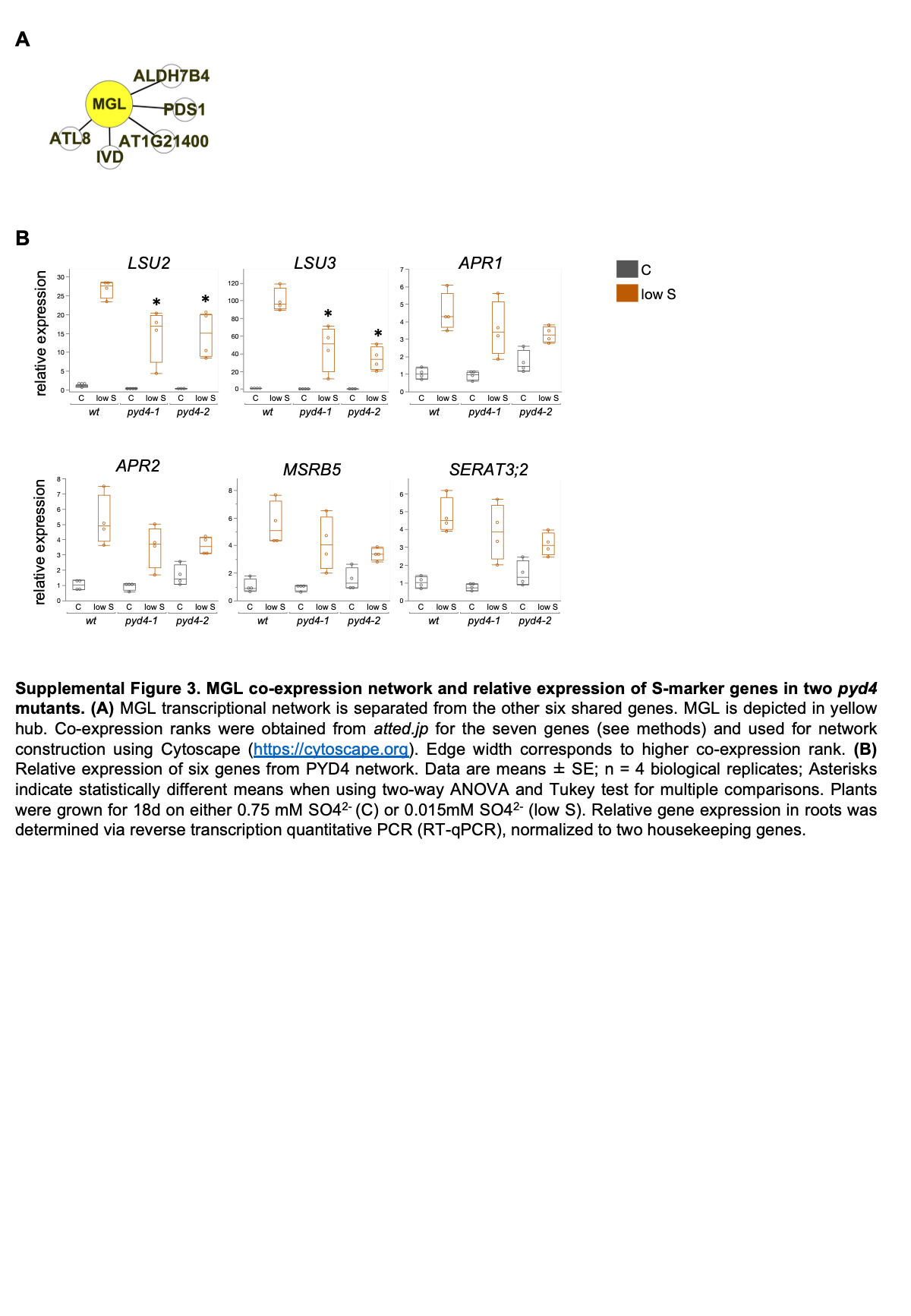

Supplement: Supplementary file 4 — Figure S3. MGL co‐expression network and relative expression of sulfate starvation marker genes in two pyd4 mutants. [file TPJ-127-0-s010.png]

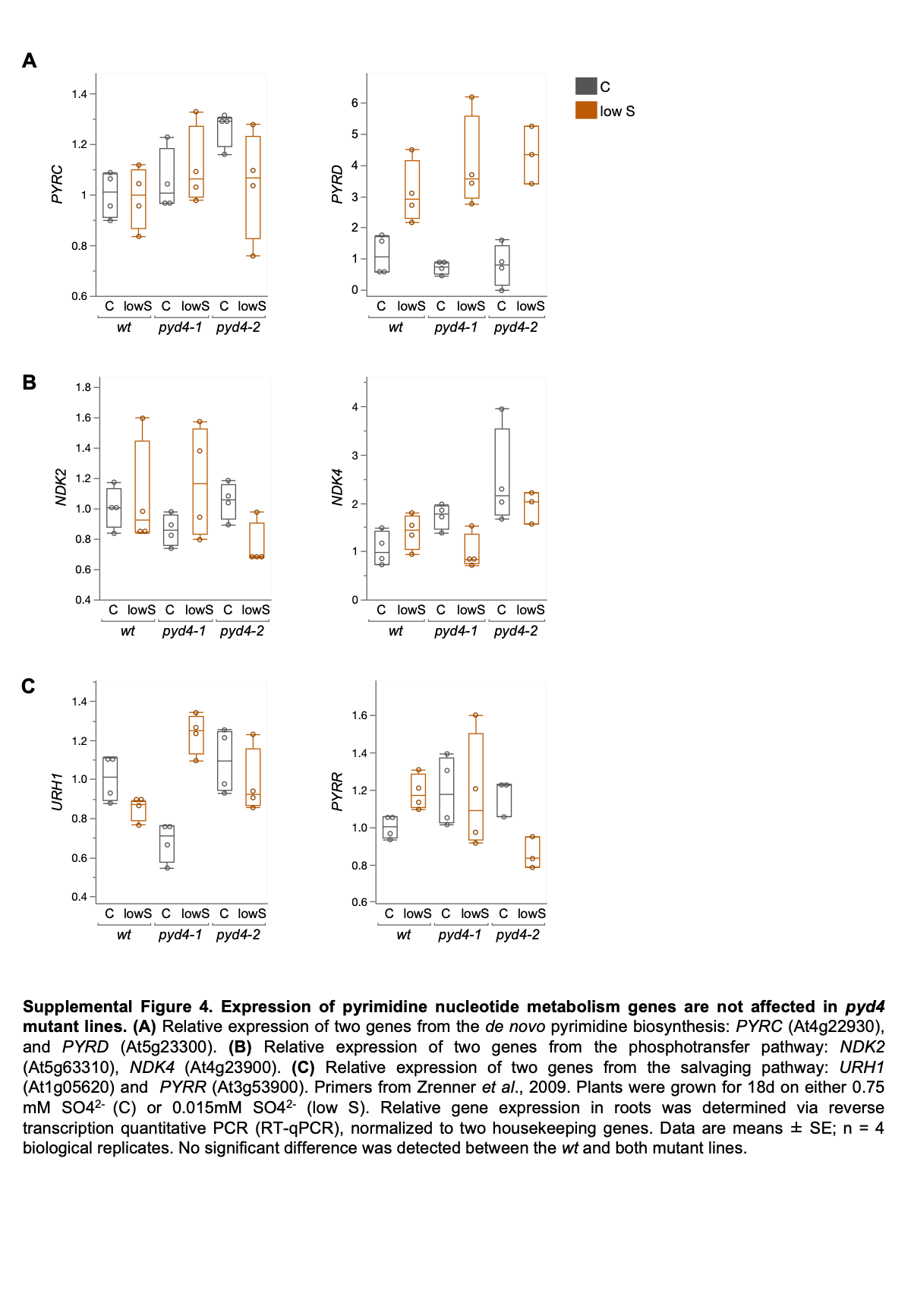

Supplement: Supplementary file 5 — Figure S4. Expression of pyrimidine nucleotide metabolism genes are not affected in pyd4 mutant lines. [file TPJ-127-0-s011.png]

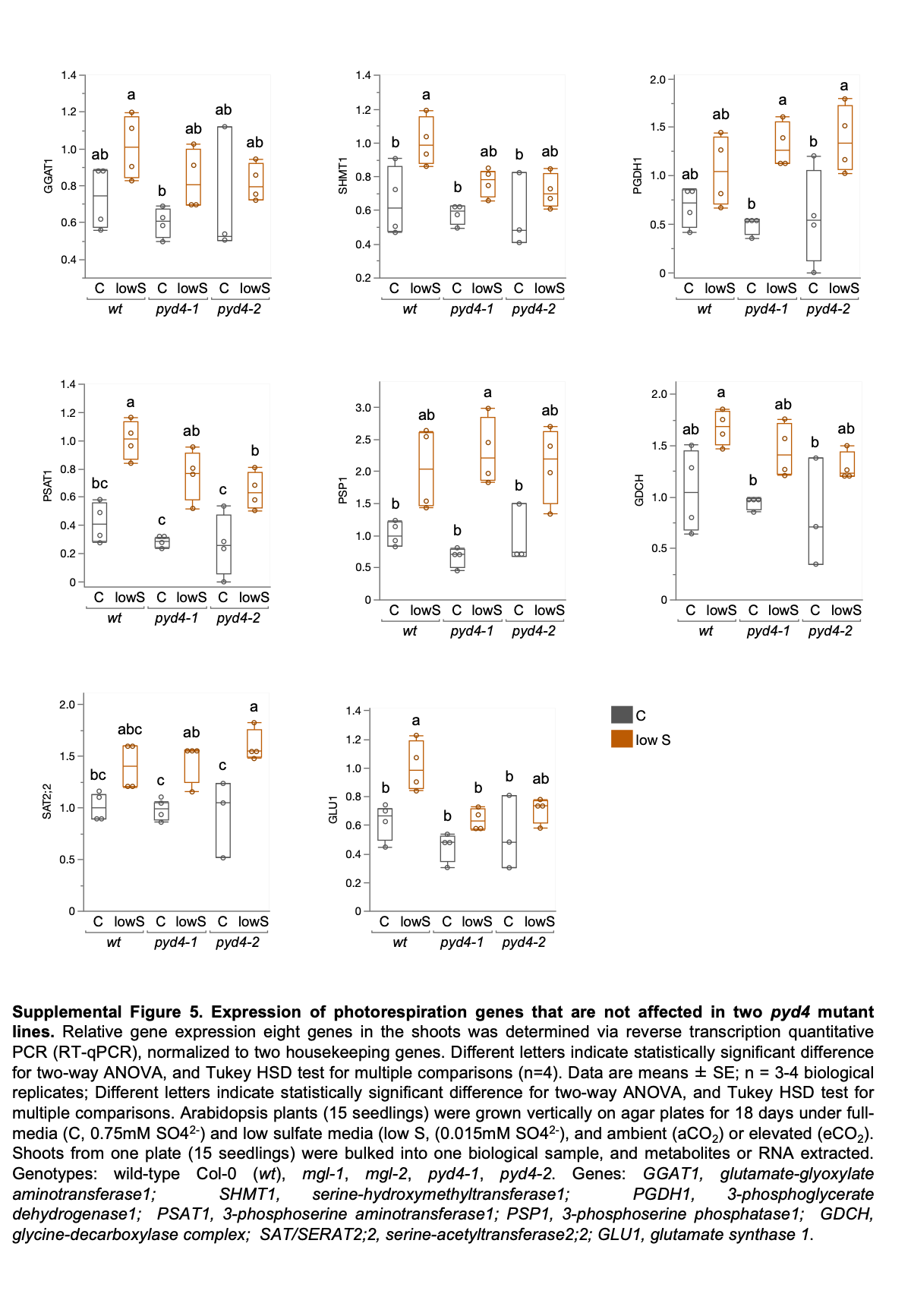

Supplement: Supplementary file 6 — Figure S5. Expression of photorespiration genes that are not affected in two pyd4 mutant lines. [file TPJ-127-0-s007.png]

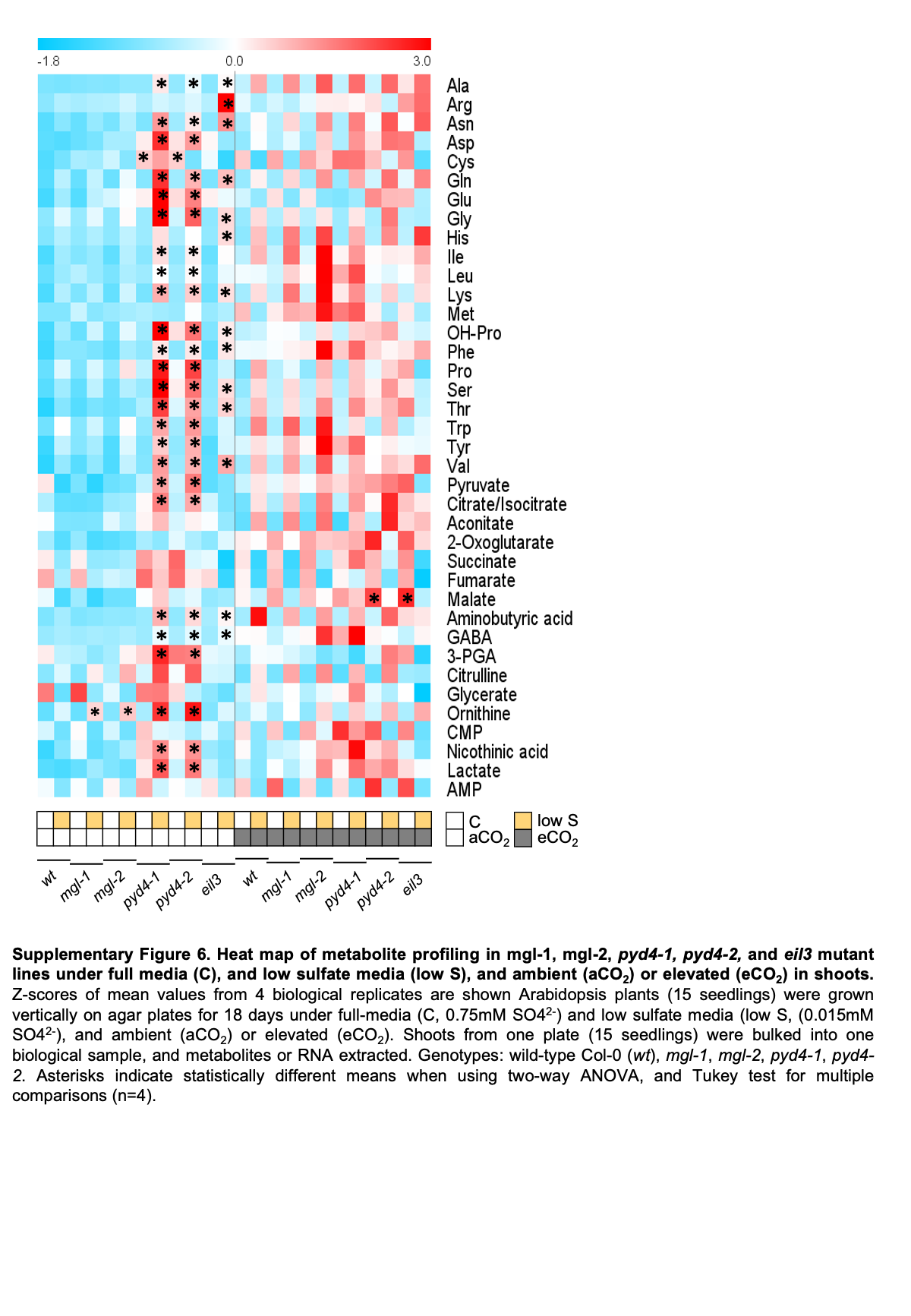

Supplement: Supplementary file 7 — Figure S6. Heat map of metabolite profiling in mgl‐1, mgl‐2, pyd4‐1, pyd4‐2, and eil3 mutant lines under full media (C), and low sulfate media (low S), and ambient (aCO2) or elevated (eCO2) in shoots. [file TPJ-127-0-s008.png]

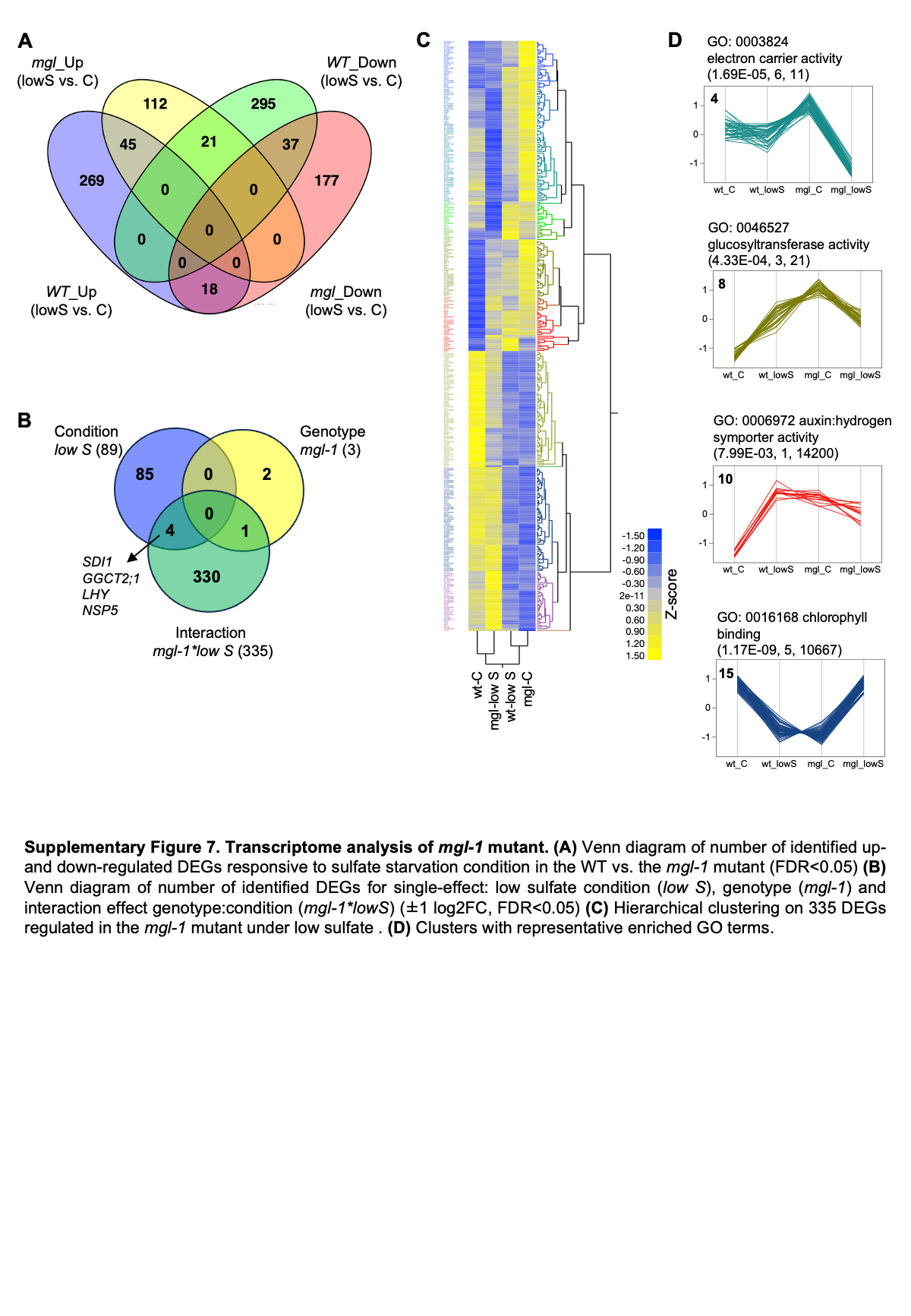

Supplement: Supplementary file 8 — Figure S7. Transcriptome analysis of mgl‐1 mutant. [file TPJ-127-0-s003.png]

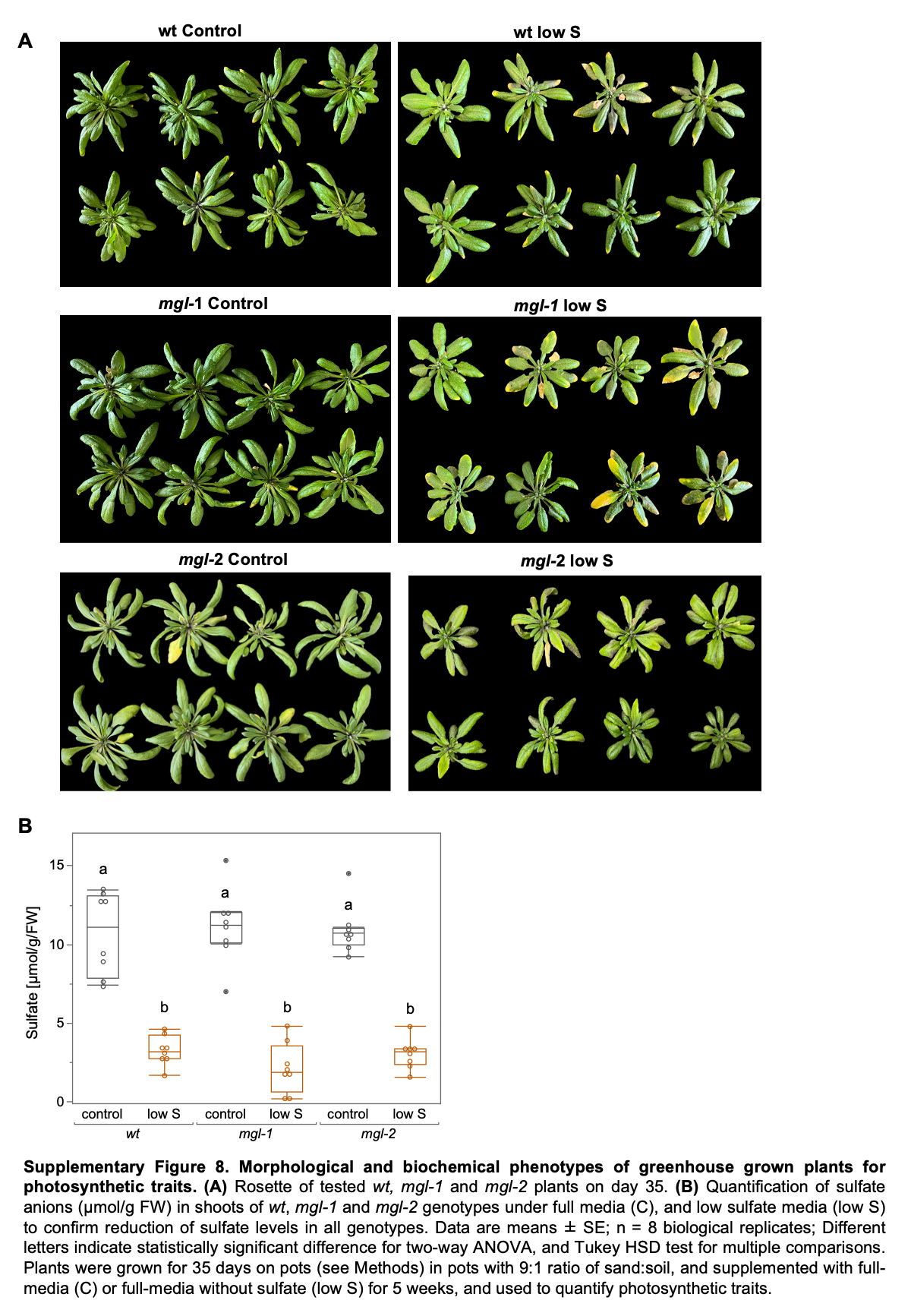

Supplement: Supplementary file 9 — Figure S8. Morphological and biochemical phenotypes of greenhouse grown plants for photosynthetic traits. [file TPJ-127-0-s009.png]

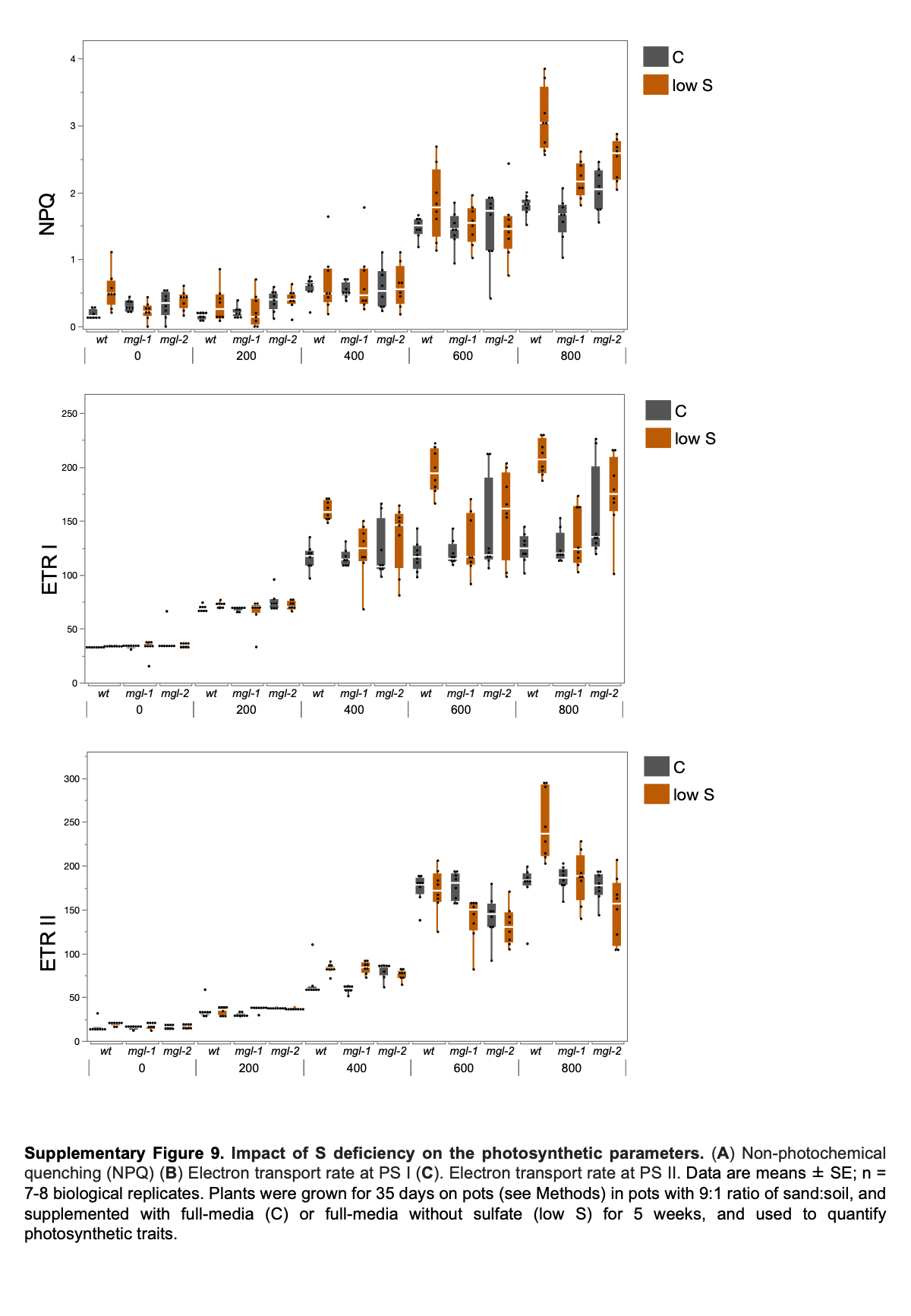

Supplement: Supplementary file 10 — Figure S9. Impact of S deficiency on the photosynthetic parameters. [file TPJ-127-0-s002.png]

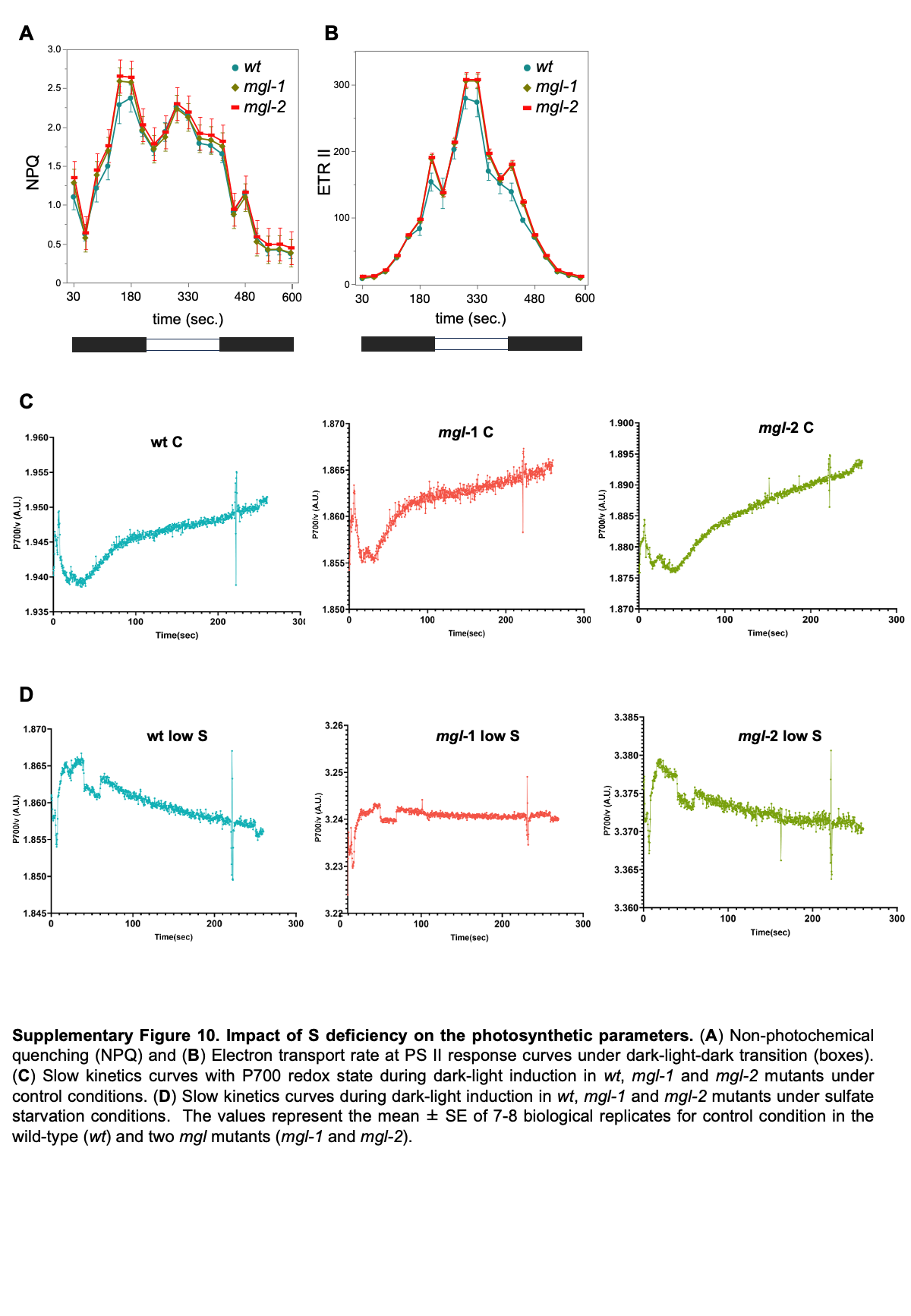

Supplement: Supplementary file 11 — Figure S10. Impact of sulfate starvation on the photosynthetic parameters. [file TPJ-127-0-s005.png]
